# Narcissism Moderates the Association Between Autonomy-Supportive Parenting and Adolescents’ Prosocial Behavior

**DOI:** 10.1007/s10964-023-01933-0

**Published:** 2023-12-26

**Authors:** Xiaoyu Lan, Chunhua Ma

**Affiliations:** 1 Promenta Research Center, Department of Psychology, University of Oslo, Oslo, Norway; 2 College of Educational Science and Technology, Northwest Minzu University, Lanzhou, China

**Keywords:** Prosocial behavior, Autonomy-supportive parenting, Narcissism, Adolescence

## Abstract

Prior research has separately investigated the associations of autonomy-supportive parenting and narcissism with adolescents’ prosocial behavior, but their joint relationships with prosocial behavior have been rarely examined. The present research aimed to expand the existing literature by scrutinizing the main and interactive associations of autonomy-supportive parenting and narcissism with adolescents’ prosocial behavior. In so doing, a series of four studies (collectively *N* = 2023), combining cross-sectional, longitudinal, and experimental designs, were conducted. The adolescents’ mean age varied from 12.42 to 15.70 years, with a balanced representation of the sexes in those studies. Converging results across four studies showed that high narcissism magnified the positive association between autonomy-supportive parenting and adolescents’ prosocial behavior. The interaction pattern presented also suggested adolescents with high narcissism scores were more affected than others—both for better *and* for worse—by autonomy-supportive parenting, although this interaction might be specific to particular facets of prosocial behavior. These results were robust after adjusting for a few key covariates and survived a set of additional analyses. The present findings provide a novel avenue to explain individual differences linking prosocial behavior with those two factors and further advance precise, individualized strategies to promote adolescents’ prosocial behavior.

## Introduction

Assisting someone without thinking about a reward or asking for anything in return is a common life experience. Such voluntary actions (e.g., sharing, helping, and cooperating) intended to help or benefit others fall under the definition of prosocial behavior (Eisenberg et al., 2015). Research has shown that prosocial behavior in adolescence is of paramount importance to both individual and societal well-being (Hui, 2022; Malti & Dys, 2018). Given the benefits of prosocial behavior, studies have suggested the important roles of autonomy-supportive parenting and adolescents’ narcissism in motivating youth to act prosocially (Donald et al., 2021; Kauten & Barry, 2016). Yet one important knowledge gap is that the combined effects of those two factors have been scarcely investigated. The present investigation aimed to extend prior scholarship by conducting a series of four studies with diverse designs and critically investigating the main and interactive associations of autonomy-supportive parenting and narcissism with adolescents’ prosocial behavior.

### Autonomy-Supportive Parenting

Autonomy-supportive parenting refers to practices where parents encourage and support their children’s autonomy by providing choices and explanations and fostering the pursuit of personal volition (Mageau et al., 2015). The current investigation used the self-determination theory as a guiding theoretical framework to study the association between autonomy-supportive parenting and adolescents’ prosocial behavior (Ryan & Deci, 2017, 2019). The self-determination theory asserts that experiences of autonomy foster prosocial behavior by strengthening the internalization of healthy social norms and bolstering adolescents’ natural tendencies toward prosocial propensities. Consistent with this theoretical proposition, empirical research has provided evidence supporting a positive association between autonomy-supportive situations and adolescents’ prosocial behavior (Donald et al., 2021). Yet research based on East Asian cultural contexts is still relatively scarce. Although the fundamental principles of self-determination theory are assumed to be universally applicable (Ryan & Deci, 2017), the interpretation and significance of perceived autonomy support may vary across different cultural contexts. Adolescents in East Asian societies, for example, often prioritize interdependence and place less emphasis on autonomy than their peers in Western societies (Markus & Kitayama, 2010). However, in the past decade, the permeation of individualistic values has led those societies to increasingly encourage autonomous characteristics, and thus parental autonomy granting might be adaptive to those societal changes (Bi et al., 2020), warranting further investigations and enriching the fundamentally universal principle of self-determination theory.

Existing empirical studies based on East Asian societies, although limited in number, have shown a positive association between autonomy-supportive parenting and adolescents’ prosocial behavior. For instance, a study on Chinese adolescents has found that autonomy-supportive parenting was positively related to adolescents’ prosocial behavior, particularly for adolescents scoring high in grit (Lan et al., 2019). More recently, research has shown a longitudinally positive relationship between autonomy-supportive parenting and adolescents’ prosocial behavior, even adjusting for the initial levels of prosocial behavior; however, this prospective association was found to be pronounced for youth manifesting high in mindfulness (Lan & Wang, 2020). Notably, these studies have mainly focused on late adolescents; in contrast, research focusing on early-to-middle adolescents who are increasingly autonomous and independent is relatively limited. Studying autonomy-supportive parenting in early-to-middle adolescence is important because parents and adolescents must work together to renegotiate the nature of parental authority and the adolescent individuation process while maintaining family connectedness (McCurdy et al., 2020). Additionally, following self-determination theory (Ryan & Deci, 2017, 2019), previous studies have emphasized significant interindividual variability in this positive association, suggesting that the manifestation of prosocial behavior often arises from the intricate interaction between situational factors and dispositional characteristics. However, individual differences in adolescents’ narcissism, which is situationally dependent and plays a significant role in their prosocial behavior (Truhan et al., 2023), have yet to be explored and present an essential area for future research.

### Narcissism

Operationalized and measured as a nonclinical personality trait in the present study, narcissism is typically characterized by grandiosity, entitlement, and the incessant need for acclaim from others (Paulhus & Williams, 2002). The study of narcissism during early-to-middle adolescence is developmentally important as the budding self-consciousness during this life period fuels adolescents’ desire to create and maintain favorable self-evaluations (Harter, 2012). Past research has exhibited a positive association between adolescents’ narcissism and prosocial behavior (Kauten & Barry, 2014, 2016), indicating that narcissistic adolescents tend to bolster their social status by actively engaging in prosocial behavior. Brunell et al. (2014), in comparison, have found that individuals who score high in narcissism tend to volunteer less for nonprofit organizations. Expanding the literature by linking narcissism with prosocial behavior is therefore important to clarify this contrasting empirical evidence.

In addition to research on the direct effect, several empirical studies have indicated that narcissism might moderate the relationship between situational variables and adolescents’ developmental outcomes.^1^ Mounting research has suggested that narcissism might equip adolescents with resilience, counteracting difficulties (Lan, 2021; Ouyang et al., 2020). For example, one study on Chinese adolescents has discovered that high narcissism buffers against the negative association of poor peer relationships with materialism (Ouyang et al., 2020). Another study has reported similar stress-buffering patterns, showing that high narcissism protects adolescents whose parents divorced from reporting increased loneliness (Lan, 2021). Yet those findings seem to conflict with Li and Ang’s (2019) results, exhibiting that high narcissism exacerbates the positive association between adolescents whose parents have had a prior arrest history and their delinquent behaviors. The above findings collectively exhibit inconsistent patterns, underscoring the need for a more comprehensive and robust investigation. Notably, one recent cross-sectional study has sought to resolve those inconsistencies and found that adolescents manifesting high narcissism show varying responsiveness to teacher autonomy support (Lan, 2023). Specifically, this study showed that adolescents tended to report the highest prosocial behavior in the presence of high teacher autonomy support and the lowest in the presence of low teacher autonomy support. Nevertheless, many lingering questions, including the causal pathway and the robustness of this association, remained unanswered in this study. The present investigation therefore aimed to expand extant research by investigating the combined effect of autonomy-supportive parenting and narcissism on adolescents’ prosocial behavior.

### Overview of Present Studies

**Fig. 1:**
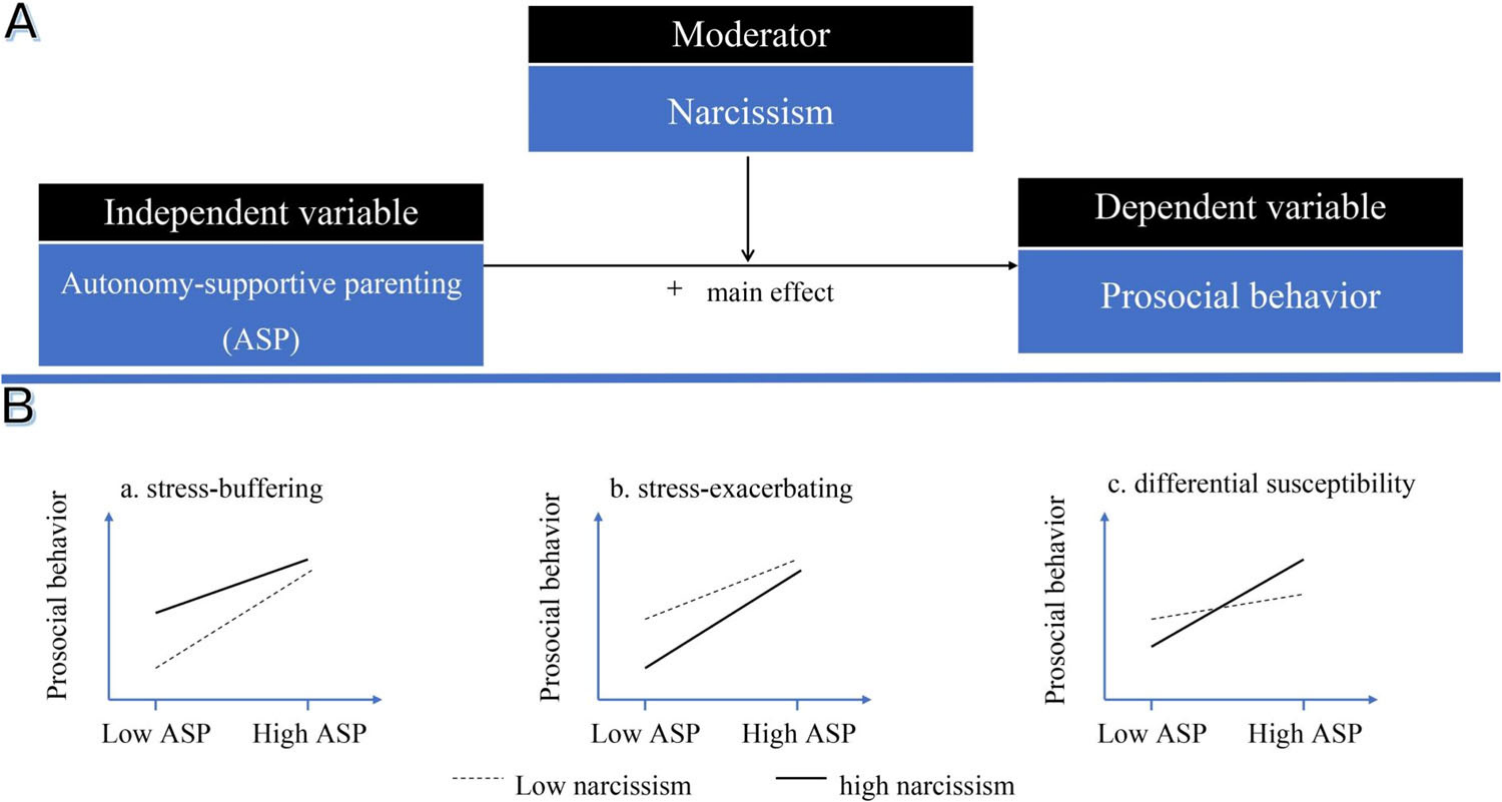
Hypothesized main and interaction effects. **A** represents the conceptual model, while **B** illustrates three plausible moderating patterns of narcissism

Guided by self-determination theory, the present studies examined the main and interactive associations of autonomy-supportive parenting and narcissism with adolescents’ prosocial behavior, as illustrated in Fig. 1A. A series of four studies, consisting of cross-sectional, longitudinal, and experimental designs, were conducted to comprehensively quantify those associations. Specifically, Study 1 preliminarily tested the associations using a cross-sectional design. Study 2 aimed to conceptually replicate those associations using different validated measurements and a large-scale sample size. Study 3 moved beyond cross-sectional designs and investigated the main and interactive relations of changes in autonomy-supportive parenting and narcissism with adolescents’ prosocial behavior after controlling for the initial levels of that behavior. Study 4 finally used an experimental manipulation task on autonomy-supportive parenting to explore the causal effect of such associations on adolescents’ prosocial behavior.

Notably, the present studies focused on conceptual rather than direct replication using identical measurements (Derksen & Morawski, 2022). Each replication study was carefully designed to introduce incremental changes while preserving the core elements of the previous study. These modifications were aimed at conducting a comprehensive robustness check, validating the stability of the study associations under diverse measurement conditions. Additionally, using those four studies with diverse sample sizes and research designs can assuage concerns that inferences are based on arbitrary or random patterns in a single data set (Lakens et al., 2018). This is especially important when the focal research question involves the interaction effect, which is often sample-specific and challenging to replicate (Sommet et al., 2023).

The four studies focused on Chinese adolescents because existing studies on both perceived autonomy support and narcissism have highly skewed toward data from Western, educated, industrialized, rich, and democratic (WEIRD) populations (Nielsen et al., 2017), producing limited generalizability. Surprisingly, limited empirical research has been grounded in East Asian cultural contexts, in which the manifestations of autonomy support (Markus & Kitayama, 2010) and positive self-views are distinctive (Boucher et al., 2009). This sampling limitation becomes significant because Chinese societies have undergone significant changes in the past decade, with individuals increasingly encouraging individualistic-oriented values and autonomous characteristics (e.g., initiative-taking and assertiveness; Xu & Hamamura, 2014; Zeng & Greenfield, 2015). Such a societal change has also been reflected in parenting practices (Bi et al., 2020) and narcissistic traits (Cai et al., 2012). Therefore, Chinese cultural contexts are well-suited to studying those associations.

Anchored in the current literature review, the following hypotheses have been formulated. First, autonomy-supportive parenting was posited to be positively associated with adolescents’ prosocial behavior (main effect; Hypothesis 1). Second, narcissism might moderate this positive association (interaction effect; Hypothesis 2). However, due to inconsistent empirical evidence on the moderating role of narcissism, three plausible interaction patterns were proposed (see Fig. 1, Panel B for illustration). First, adolescents scoring high in narcissism might be more resistant to difficulties associated with low autonomy-support parenting, reporting high prosocial behavior (stress-buffering hypothesis; Hypothesis 2a). Alternatively, adolescents scoring high in narcissism might be especially at risk of low autonomy-supporting parenting, thus exhibiting low prosocial behavior (stress-exacerbating hypothesis; Hypothesis 2b). Finally, adolescents manifesting high narcissism might be more susceptible than others to both the positive and the negative effects of autonomy-supportive parenting (differential susceptibility hypothesis; Hypothesis 2c). In general, due to the intended universality of the self-determination theory, those hypotheses were expected to be replicated in the four studies with diverse sample sizes, measures, and research designs.

When examining those hypotheses, the present studies aimed to gather robust and incremental estimates by including several control variables. Building upon previous research into the prosocial behavior of Chinese adolescents (Zhou et al., 2022), the present studies considered key sociodemographic covariates, such as adolescents’ age, sex, the educational level of their parents, and family wealth, to isolate their associations with prosocial behavior. By doing so, it becomes possible to estimate the incremental variances attributed to the focal variables under investigation. In addition, the same research procedures and data analytical plans (including missing data handling technique) were adopted, unless otherwise noted, to minimize the potential “noise” between different studies. Any similarities and differences observed could therefore be mainly attributed to the effect of study associations.

## Study 1

To examine the hypothesized associations, a cross-sectional design with a modest sample size was first utilized to probe the moderating role of narcissism in the association between autonomy-supportive parenting and adolescents’ prosocial behavior.

## Method of Study 1

### Participants and Procedure

During regular school hours, adolescents whose parents or legal guardians provided consent were instructed to complete an anonymous, questionnaire-based survey under the supervision of trained graduate students. The study procedures were approved by the Institutional Review Board at the Northwest Minzu University and school authorities in China before data collection. In Study 1, conducting an a priori power analysis was challenging because the expected effect size of the interaction relied on the “shape” of the interaction, which was hypothesized to be exploratory in this research project. The researchers thus adopted a more flexible sample size plan.^2^ Specifically, a priori power analysis using G*power (Faul et al., 2007) was conducted for the main effect only (*N*_minimum_ = 103 with 80% statistical power) to have the lowest boundary of the required sample size. The small-to-medium effect size was employed in this power analysis based on prior meta-analytical findings (Thielmann et al., 2020; Vasquez et al., 2016). Subsequently, the researchers consulted prior research investigating similar constructs using linear regression to guide the sample size plan (Li et al., 2023).

In total, a convenience sample of 318 secondary school students (*M*_age_ = 12.91) volunteered to participate in Study 1.^3^ The sample had slightly more girls (52.5%) than boys. More details regarding participants’ family backgrounds can be viewed in the supplementary materials (Table S1).

### Measures

All self-reported, Chinese-administrated measurements were carefully selected based on existing psychometric properties. The brevity and simplicity of the measurements were prioritized to decrease participation burdens, given that completing relevant tasks with long formats might be difficult for young adolescents (Gogol et al., 2014).

### Prosocial Behavior

Prosocial behavior was assessed using a subscale from the Strengths and Difficulties Questionnaire (Goodman et al., 1997; Liu et al., 2013). This subscale has five items (e.g., “I am helpful if someone is hurt, upset, or feeling ill”), and each was assessed on a 3-point scale varying from 0 (*not true*) to 2 (*certainly true*). The summed scores were computed, and higher scores indicated higher prosocial behavior. In Study 1, this subscale had borderline acceptable internal consistency (Cronbach’s alpha = 0.65 and McDonald’s omega = 0.65) but was consistent with prior research on Chinese adolescents (Teuber et al., 2022).

### Autonomy-Supportive Parenting

Autonomy-supportive parenting was assessed using the Perceived Parental Autonomy Support Scale, a measure culturally adapted by Wang et al. (2007). This questionnaire includes 16 items, separated by the father’s and mother’s dimensions (e.g., “My father/mother is willing to consider issues from my perspective”). All items were scored on a 5-point scale running from 1 (*strongly disagree*) to 5 (*strongly agree*). The average scores were calculated, with higher scores indicating a greater perception of autonomy-supportive situations provided by both parents. This scale had good internal consistency in Study 1 (Cronbach’s alpha = 0.89 and McDonald’s omega = 0.89).

### Narcissism

Narcissism was measured using the Childhood Narcissism Scale (Thomaes et al., 2008), a brief assessment demonstrating good psychometric properties in Chinese adolescents (Xu et al., 2020). This scale contains ten items (e.g., “I am a very special person”), rated on a 4-point scale varying from 1 (*strongly disagree*) to 4 (*strongly agree*). Average scores were calculated, with higher scores indicating greater narcissism. In Study 1, this scale had adequate internal consistency (Cronbach’s alpha = 0.79 and McDonald’s omega = 0.79).

### Covariates

Study 1 controlled for adolescents’ age, sex, highest education levels of their parents, and family wealth. Sex was dummy-coded, with 0 representing girls and 1 representing boys. Regarding the measurement of parental education levels, adolescents responded to two items, one for the father and another for the mother, represented by three categories (*1*-*middle school or lower*, *2*-*high school*, and *3*-*undergraduate education or higher*). The scores across those items were combined into a composite score, with a higher score indicating a higher parental education level. Since young adolescents often have difficulties accurately reporting family income, resulting in a high non-response rate, a four-item family affluence scale was employed (Boyce et al., 2006) as a proxy reflecting the common features of family wealth. A summed score was created for this scale, with higher scores indicating higher family wealth.

### Data Analytical Plan

Leveraging against R software (R Core Team, 2022), data analyses were first conducted by summary statistics and followed by bivariate correlations using the R package *corrplot* (Wei & Simko, 2017). Summary statistics present the means, standard deviations, skewness and kurtosis values, and internal consistency for the variables. Since only a small number of missing values (less than 1%) appeared in Study 1, the researchers replaced the missing values with an expectation-maximization algorithm after confirming Little’s (1988) missing completely at random test.

To examine research hypotheses, a hierarchical multiple regression was performed, in which covariates were entered in Step 1, main effects in Step 2, and the interaction in Step 3. Notably, this analysis was conducted using the Ordinary Least Squares method. Before conducting this regression, multicollinearity was examined using the variance inflation factor. The results showed that all predictors were lower than 2. The significant interaction term was further assessed with simple slope analyses, visualized for the moderator between −2 and 2 standard deviations using the R package *InterActive* (McCabe et al., 2018). This range was selected because it generally represents the variable’s observed range. To provide more comprehensive information for the interaction, the Johnson-Neyman technique was also performed using the R package *interactions* (Long, 2022) to inspect the regions of significance. This technique complemented simple slope analyses by providing a full range of the moderator affecting the study association from statistically non-significant to significant (Lin, 2020). Following the guidelines outlined by Green (2010), the interaction’s effect size was interpreted as small, medium, and large, corresponding to R² values of 0.008, 0.07, and 0.19.^4^

### Additional Analyses

A series of additional analyses were conducted to examine the robustness of the research findings. First, analyses were conducted by separating father and mother autonomy support because they might play a differential role in adolescents’ prosocial behavior (Vrolijk et al., 2020). Second, analyses were conducted using Poisson regression to evaluate whether the main and interaction effects were replicable. This type of regression was performed considering that a self-reported ordinal scale measured prosocial behavior in Study 1 and that the summed scores of this scale generated ordinal (instead of continuous) data. Prior research has indicated that analyzing an ordinal outcome while assuming its continuity might distort estimates of effect sizes and inflate false positive rates (Rohrer & Arslan, 2021). Third, missing data were handled with regression-based multiple imputations (Enders, 2022). One hundred imputed datasets were generated, and the pooled parameter estimates were obtained according to Rubin’s (1987) recommendations. This extended analysis was done to ensure the robustness of the findings under different imputation methods. Finally, a post hoc power analysis would be implemented to estimate the statistical power associated with the current sample size, considering the specific shape of the interaction and the magnitude of the effect size. When performing those additional analyses, the previously mentioned covariates were also controlled.

## Results of Study 1

### Summary Statistics and Bivariate Correlations

**Fig. 2:**
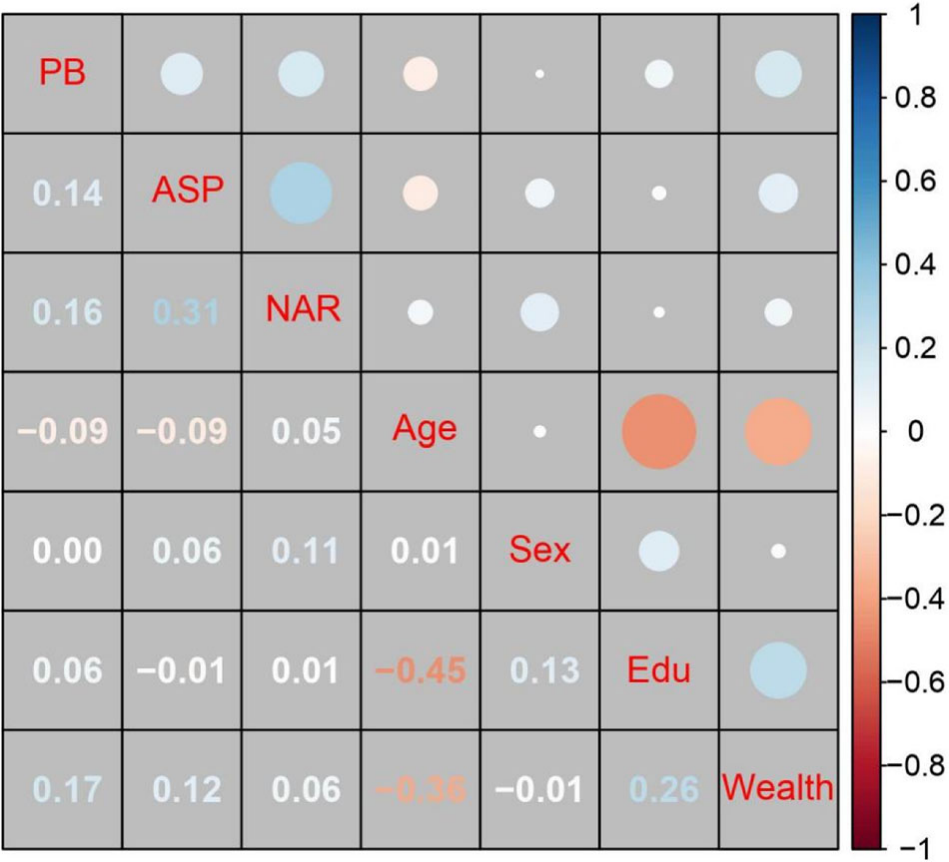
Correlation matrix in Study 1 (*N* = 318). Numbers and circles in blue font signify positive associations, while those in red indicate negative ones. More saturated colors and larger circles reflect stronger correlations. The descriptive statistics and the raw correlation matrix can be found in Table S2. Sex was coded as 0 = girls and 1 = boys. PB prosocial behavior, ASP autonomy-supportive parenting, NAR narcissism, and Edu parental education level. ^*^p-value at a 0.05 level reached significance when *r* coefficients were more than 0.11

Summary statistics and bivariate correlations are all presented in Table S2 for the sake of space limitation. As shown in Fig. 2, autonomy-supportive parenting, narcissism, and prosocial behavior were all positively correlated.

### Hierarchical Regression Analysis Predicting Prosocial Behavior

Table 1 presents the results of the regression analysis in Study 1. In total, the model explained an 8% variance in prosocial behavior. The main effects examined in the second step showed that narcissism was positively related to prosocial behavior, whereas autonomy-supportive parenting was not. The first hypothesis was thus not supported. In Step 3, the two-way interaction was significant, further explaining the 2% variance. The effect size of this interaction was small to medium.

**Table 1:** Hierarchical regression analysis predicting prosocial behavior in Study 1 (*N* = 318)

	*b*	*b SE*	95% CI for *b*		*β*	*t*	*p*	*R*^2^	△*R*^2^	△*F*
Step 1										
Age	−0.04	0.08	−0.19	0.12	−0.03	−0.45	0.65			
Sex^a^	−0.01	0.22	−0.44	0.43	0.00	−0.03	0.98			
Parental education	0.01	0.10	−0.19	0.21	0.00	0.07	0.94			
Family wealth	0.15	0.06	0.04	0.26	0.16	2.67	0.01	0.03	0.03	2.47^*^
Step 2										
Age	−0.04	0.08	−0.19	0.12	−0.03	−0.50	0.62			
Sex	−0.09	0.22	−0.52	0.34	−0.02	−0.41	0.68			
Parental education	0.02	0.10	−0.18	0.22	0.01	0.18	0.86			
Family wealth	0.13	0.06	0.02	0.24	0.14	2.35	0.02			
Autonomy-supportive parenting	0.18	0.13	−0.08	0.44	0.08	1.34	0.18			
Narcissism	0.50	0.22	0.07	0.92	0.13	2.31	0.02	0.06	0.03	5.01^**^
Step 3										
Age	−0.05	0.08	−0.20	0.10	−0.04	−0.63	0.53			
Sex	−0.04	0.22	−0.46	0.39	−0.01	−0.17	0.86			
Parental education	−0.02	0.10	−0.22	0.18	−0.01	−0.20	0.84			
Family wealth	0.14	0.06	0.04	0.25	0.16	2.62	0.01			
Autonomy-supportive parenting	−1.23	0.51	−2.24	−0.22	0.11	−2.40	0.02			
Narcissism	−1.65	0.79	−3.21	−0.10	0.13	−2.10	0.04			
Autonomy-supportive parenting X Narcissism	0.60	0.21	0.18	1.02	0.14	2.84	0.01	0.08	0.02	8.05^**^

*p < 0.05, **p < 0.01

^a^coded as 0 = girls and 1 = boys

**Fig. 3:**
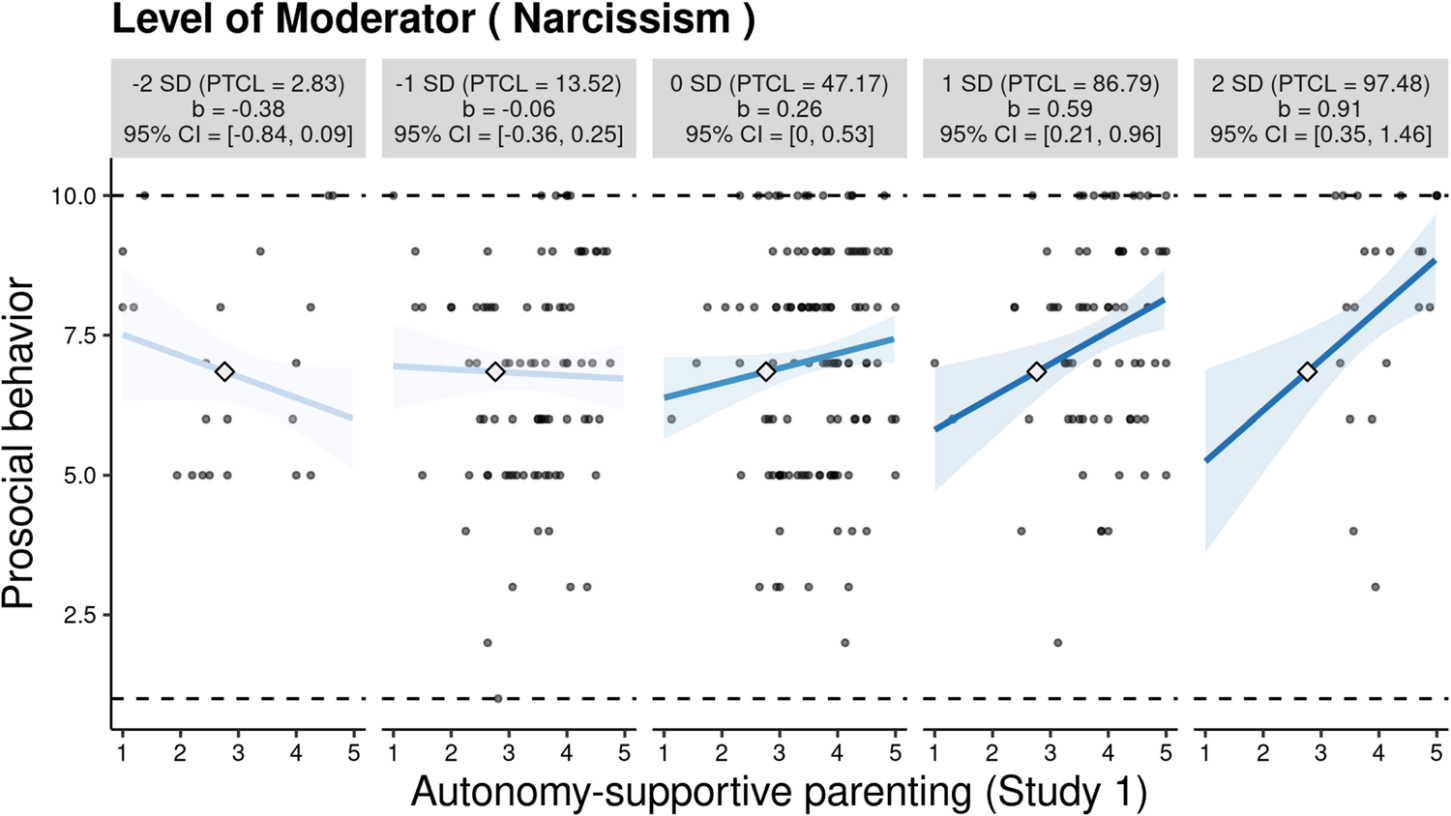
The moderating role of narcissism in the association between autonomy-supportive parenting and adolescents’ prosocial behavior in Study 1 (*N* = 318). Each graph displays the 95% confidence interval (CI) as a shaded area, the empirical data as gray circles, the maximum and minimum values of prosocial behavior as dashed horizontal lines, and the crossover point as a diamond. The x-axes represent the full range of autonomy-supportive parenting. PTCL percentile

Figure 3 illustrates the patterns of this significant interaction, showing that high narcissism amplified the positive association between autonomy-supportive parenting and adolescents’ prosocial behavior. Specifically, autonomy-supportive parenting showed a positive correlation with prosocial behavior at one and two standard deviations above the mean of narcissism. Conversely, at the mean level of narcissism, as well as one and two standard deviations below the mean, this association became non-significant, exhibiting a relatively flat slope. The examination of regions of significance, as illustrated in Fig. S1, showed that autonomy-supportive parenting was positively associated with prosocial behavior in adolescents with 0.05 standard deviations above the mean in narcissism. Additionally, 41.82% of observations in narcissism fell within this region of significance.

Additional steps on this interaction pattern should be taken because a crossover point is shown in Fig. 3. From a descriptive point of view, in the presence of high autonomy-supportive parenting, adolescents presenting with higher (versus lower) narcissism reported a higher intercept for prosocial behavior. In contrast, in the presence of low autonomy-supportive parenting, adolescents with higher narcissism reported a lower intercept for prosocial behavior. This interaction pattern seemingly supported the differential susceptibility hypothesis (Hypothesis 2c). Confirmatory analysis was conducted following point and interval estimates of the crossover point (Widaman et al., 2012). The results showed that the crossover point (*C* = 2.76, *SE* = 0.46) and the interval estimate (95% CI = [1.65, 3.67]) fell within the observed range of autonomy-supportive parenting in Study 1. The differential susceptibility hypothesis of narcissism was thus confirmed.

### Additional Analyses

First, the results separating father and mother autonomy support, as reported in Tables S3 and S4 and Figs. S2 and S3, still substantially resembled those with a combined single-score analysis. Second, the results based on Poisson regression converged on the same conclusions as the linear regression, as shown in Table S5. Thus, the original analyses with the combined score of autonomy-supportive parenting and linear regression were retained, and the same analyses were discontinued in the subsequent studies. Third, as detailed in Table S6, the results using multiple imputations were largely consistent with those obtained using the single imputation method. While multiple imputations offer notable advantages over single imputation, the data exhibited a missing data pattern that adhered to the missing completely at random assumption, with a low percentage of missingness. Considering the computational convenience of single imputation, as emphasized by Javanbakht et al. (2022), the original choice of using the single imputation method for the analyses was maintained. Finally, according to the generated interaction shape and identified effect size, a post hoc power analysis showed that the current sample size could yield approximately 80% statistical power with a two-tailed test (Sommet et al., 2023).

## Brief Discussion of Study 1

The findings from Study 1 offer preliminary insights into the study associations. The main effect hypothesis was not supported, but the findings gave an initial indication that high narcissism enhanced the positive association between autonomy-supportive parenting and adolescents’ prosocial behavior. The inspection of the crossover point also considered narcissism as a differential susceptibility trait in this positive association. Notably, several limitations should be considered in Study 1. First, Study 1 contained a modest sample size, and the overall model explained a relatively weak variance in prosocial behavior. Second, concerns related to social desirability, when studying the correlates of prosocial behavior, were not statistically controlled. Finally, all the scales were based on self-reported questionnaires, potentially inflating the study associations. Study 2 was thus conducted to combat those limitations and justify the robustness of those findings.

## Study 2

Using a large-scale sample size, Study 2 was designed to conceptually replicate the findings obtained in the first study using different validated scales of prosocial behavior and narcissism. In addition, socially desirable responses were adjusted using a well-validated scale, and parent reports on their highest educational level and family income were gathered to ease the concerns of common method contamination.

## Methods of Study 2

### Participants and Procedure

In Study 2, a priori power analysis was not performed because data collection was conducted based on a large school collaboration project in which adolescents from (almost) entirely public schools voluntarily participated. A large-scale sample size in this regard would ensure sufficient statistical power to perform the subsequent analyses (Sommet et al., 2023). Participants in Study 2 were convenience-based and totaled 2098 adolescents, with an average age of 15.70 years.^5^ The sample contained slightly fewer girls (44.1%) than boys. Participants’ family backgrounds can be viewed in Table S1.

### Measures

#### Prosocial Behavior

Prosocial behavior^6^ was measured by a culturally sensitive, multidimensional scale developed by Yang, Zhang, and Kou (2016). This scale comprises 21 items and distinguishes four specific types of prosocial behavior, which include: altruistic behavior (six items; Cronbach’s alpha = 0.80; McDonald’s omega = 0.80), primarily driven by responding to others’ needs (e.g., “When I see others in difficulty, I will proactively offer help”); behavior benefiting public welfare (five items; Cronbach’s alpha = 0.76; McDonald’s omega = 0.76), largely motivated by conforming to social norms (e.g., “I like participating in social activities for the public good”); relational behavior (five items; Cronbach’s alpha = 0.72; McDonald’s omega = 0.72), motivated by maintaining harmonious relationships within one’s community (e.g., “I would like to invite other bystanders to join in our games”); trait prosociality (five items; Cronbach’s alpha = 0.76; McDonald’s omega = 0.76), where individuals demonstrate positive character traits to enhance self-esteem and maintain a favorable social standing (e.g., “I think that one of the best things about helping others is that it makes me look good”). All items were rated on a 7-point scale (1 = *strongly disagree*; 7 = *strongly agree*), and an average score across all items was created to represent a global score of prosocial behavior, with higher values indicating greater prosocial behavior. Compared with the prosocial behavior measurement used in Study 1, the scale in Study 2 demonstrated much improved internal consistency (Cronbach’s alpha = 0.84 and McDonald’s omega = 0.85).

#### Autonomy-Supportive Parenting

Autonomy-supportive parenting was measured using the same scale as Study 1. Nevertheless, based on the additional analyses conducted in the first study, the overall dimension of autonomy-supportive parenting rather than separated by each parent was used. The internal consistency of this scale in Study 2 was as good as that of Study 1 (Cronbach’s alpha = 0.90 and McDonald’s omega = 0.90).

#### Narcissism

A subscale of the Short Dark Triad developed by Jones and Paulhus (2014) and validated by Zhang et al. (2019) was used to assess narcissism. This 9-item subscale (e.g., “People see me as a natural leader”) was scored on a 5-point Likert-type scale running from 1 (*strongly disagree*) to 5 (*strongly agree*). A composite narcissism score by averaging the nine items was created. Higher scores indicated greater narcissism. In line with prior research (Zhang et al., 2019), this subscale had adequate internal consistency (Cronbach’s alpha = 0.70 and McDonald’s omega = 0.72).

#### Covariates

When giving informed consent, parents were asked to indicate their educational background and family monthly income. Social desirability in Study 2 was measured via the 16-item social desirability scale from Schuessler et al. (1978), rated by a 7-point Likert scale. Mean scores were created, and higher scores indicated higher social desirability. For the social desirability scale used in Study 2, Cronbach’s alpha was 0.87, and McDonald’s omega was 0.87.

#### Additional Analyses

The first additional analysis incorporated teacher autonomy support in the model. This analysis aimed to examine the contextual specificity of the main and interaction effects, given that teachers are important socialization agents extensively influencing school-aged adolescents’ manifestations of prosocial behavior (Streit et al., 2023). This additional analysis was also informed by prior research (Lan, 2023), showing that teacher autonomy support interacted with adolescents’ narcissism to predict prosocial behavior. Second, given that prosocial behavior captures a variety of underlying motivations and behaviors (Pfattheicher et al., 2022), the findings based on a global assessment of prosocial behavior might not be generalizable when taking the specificity of such behaviors into account. It is worth noting that Study 2 incorporated a multidimensional assessment of prosocial behavior, allowing for a comprehensive examination of the associations within this study.

## Results of Study 2

### Summary Statistics and Bivariate Correlations

**Fig. 4:**
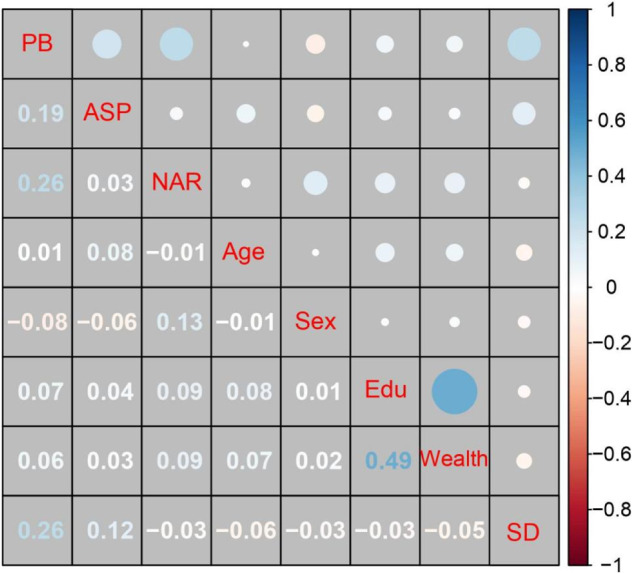
Correlation matrix in Study 2 (*N* = 2098). Numbers and circles in blue font signify positive associations, while those in red indicate negative ones. More saturated colors and larger circles reflect stronger correlations. The descriptive statistics and the raw correlation matrix can be seen in Table S6. Sex was coded as 0 = girls and 1 = boys. PB prosocial behavior, ASP autonomy-supportive parenting, NAR narcissism, Edu parental education level, and SD social desirability. ^*^p-value at a 0.05 level reached significance when *r* coefficients were more than 0.05

Summary statistics and correlations between all variables appear in Table S7. As illustrated in Fig. 4, the more autonomy-supportive parenting and narcissism the participants reported, the more prosocial behavior they tended to show. Notably, social desirability was also positively associated with prosocial behavior and autonomy-supportive parenting.

### Hierarchical Regression Analysis Predicting Prosocial Behavior

Table 2 shows the results of the regression analysis in Study 2. In total, the model explained an 18% variance in prosocial behavior. Regarding the main effects tested in the second step, both autonomy-supportive parenting and narcissism were positively related to prosocial behavior, accounting for an additional 9% variance in prosocial behavior. The first hypothesis was hence supported. In addition to the main effects, the interaction effect examined in the third step reached significance and helped explain a 1% variance. Consistent with the first study, the effect size of this interaction was small to medium. The second hypothesis was again supported.

**Table 2:** Hierarchical regression analysis predicting prosocial behavior in Study 2 (*N* = 2098)

	*b*	*b SE*	95% CI for *b*		*β*	*t*	*p*	*R*^2^	△*R*^2^	△*F*
Step 1										
Age	0.01	0.01	−0.02	0.03	0.01	0.59	0.55			
Sex^a^	0.13	0.04	0.06	0.20	0.08	3.63	<0.001			
Parental education	0.04	0.02	0.00	0.07	0.05	2.10	0.04			
Family wealth	0.04	0.02	0.00	0.07	0.05	1.94	0.05			
Social desirability	0.66	0.05	0.56	0.77	0.26	12.39	<0.001	0.08	0.08	36.31^***^
Step 2										
Age	0.00	0.01	−0.02	0.02	0.01	0.35	0.73			
Sex	0.17	0.03	0.10	0.23	0.10	5.00	<0.001			
Parental education	0.02	0.02	−0.01	0.05	0.03	1.32	0.19			
Family wealth	0.02	0.02	−0.01	0.06	0.03	1.24	0.22			
Social desirability	0.63	0.05	0.53	0.73	0.25	12.32	<0.001			
Autonomy-supportive parenting	0.17	0.02	0.12	0.21	0.15	7.27	<0.001			
Narcissism	0.33	0.02	0.28	0.37	0.27	13.16	<0.001	0.17	0.09	117.38^***^
Step 3										
Age	0.00	0.01	−0.02	0.02	0.01	0.29	0.77			
Sex	0.17	0.03	0.11	0.24	0.10	5.16	<0.001			
Parental education	0.02	0.02	−0.01	0.05	0.03	1.33	0.18			
Family wealth	0.02	0.02	−0.01	0.06	0.03	1.38	0.17			
Social desirability	0.62	0.05	0.52	0.72	0.24	12.21	<0.001			
Autonomy-supportive parenting	−0.30	0.10	−0.49	−0.10	0.13	−3.03	0.00			
Narcissism	−0.25	0.12	−0.48	−0.01	0.26	−2.05	0.04			
Autonomy-supportive parenting X Narcissism	0.15	0.03	0.09	0.21	0.09	4.88	<0.001	0.18	0.01	23.77^***^

***p < 0.001

^a^coded as 0 = girls and 1 = boys

**Fig. 5:**
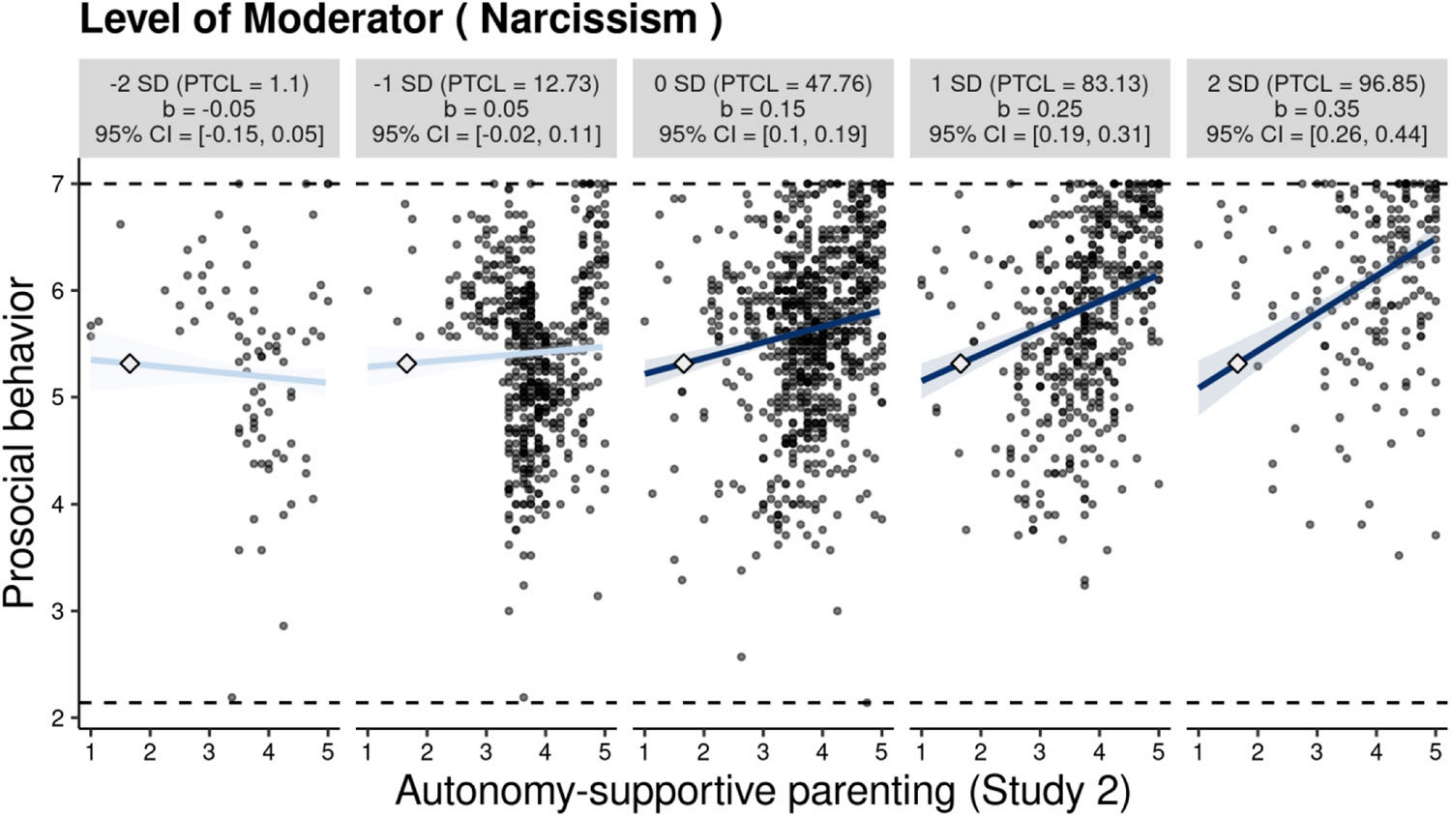
The moderating role of narcissism in the association between autonomy-supportive parenting and adolescents’ prosocial behavior in Study 2 (*N* = 2098). *Note*. Each graph displays the 95% confidence interval (CI) as a shaded area, the empirical data as gray circles, the maximum and minimum values of prosocial behavior as dashed horizontal lines, and the crossover point as a diamond. The x-axes represent the full range of autonomy-supportive parenting. PTCL percentile

Similar to those reported in the first study, in Study 2, high narcissism enhanced this positive association (see Fig. 5). Specifically, a positive correlation was observed between autonomy-supportive parenting and prosocial behavior at the mean, as well as at one and two standard deviations above the mean level of narcissism. However, this association was not significant at one and two standard deviations below the mean level of narcissism. As shown in Fig. S4, autonomy-supportive parenting demonstrated a positive association with prosocial behavior in the region at −0.85 standard deviations above the mean in narcissism, including 78.79% of the observations.

However, the inspection of the crossover point did not support the differential susceptibility pattern identified in the first study. Statistically, the point estimate (*C* = 1.67, *SE* = 0.48) was within the observed range of autonomy-supportive parenting in Study 2, but the lowest boundary of this point (95% CI = [0.72, 2.59]) fell outside the observed range. Instead, the interaction pattern seemed to support “vantage sensitivity” (Pluess & Belsky, 2013), in which high narcissism amplified the positive association of autonomy-supportive parenting on adolescents’ prosocial behavior, particularly in the context of high autonomy-supportive parenting, but not that of low autonomy-supportive parenting.

### Additional Analyses

First, the results exhibited, as shown in Table S8, teacher autonomy support was more strongly linked to adolescents’ prosocial behavior than autonomy-supportive parenting. However, the interaction effect between teacher autonomy support and narcissism was not significant, whereas the interaction between autonomy-supportive parenting and narcissism remained significant. The results suggested that teacher autonomy support might facilitate adolescents’ prosocial behavior as well, but the moderating role of narcissism seemed contextually specific to autonomy-supportive parenting. Second, as reported in Tables S9–S12 and the associated Figs. S5–S8, when taking specific dimensions of prosocial behavior into account, the subscales of altruistic behavior, public good, and trait prosociality exhibited similar findings to those with a unidimensional score of prosocial behavior. The inspection of the point and interval estimates showed that the lowest boundaries of 95% CI fell outside of the observed range of autonomy-supportive parenting (*C* = 0.86, *SE* = 0.91, 95% [−0.92, 2.64] for altruistic behavior; *C* = 1.89, *SE* = 0.46, 95% [0.98, 2.80] for public good; *C* = 1.61, *SE* = 0.64, 95% [0.35, 2.87] for trait prosociality). However, with regard to relational behavior subscale, adolescents manifesting high narcissism in Study 2 seemed more susceptible than others to being affected by autonomy-supportive parenting, for better *and* for worse. The point and interval estimate also supported the differential susceptibility pattern (*C* = 2.11, *SE* = 0.32, 95% [1.48, 2.73]), as identified in the first study.

## Brief Discussion of Study 2

The second study showed a significant main effect between autonomy-supportive parenting and adolescents’ prosocial behavior, and high narcissism enhanced this positive association. Nevertheless, the differential susceptibility pattern of narcissism was partially replicated for the subscale of relational behavior only. For the global score of prosocial behavior and the other three subscales, the interaction pattern supported vantage sensitivity (Pluess & Belsky, 2013), in which high narcissism enhanced this positive association, particularly in the context of high autonomy-supportive parenting. This finding suggests that narcissistic adolescents are responsive to parental autonomy support, with these responses varying depending on the subtype-specific of prosocial behavior. The second study, armed with an adequately powered sample size, unfortunately, did not fully confirm the findings identified in the first study. Future research is still needed to clarify these inconsistent results. Additionally, Studies 1 and 2 focused on “static” and concurrent estimates. This limitation merits further examination using a longitudinal design because parenting practices are dynamic during adolescence (Zheng & McMahon, 2022). Therefore, Study 3 was implemented by moving beyond cross-sectional designs to examine how those main and interactive associations unfolded over time.

## Study 3

Using a two-wave longitudinal design spanning one year, Study 3 aimed to estimate the main and interactive associations of change in autonomy-supportive parenting and narcissism with adolescents’ prosocial behavior after accounting for the baseline of prosocial behavior.

## Methods of Study 3

### Participants and Procedure

The investigation in Study 3 was conducted in the middle of two consecutive academic years, sampled one year apart. In the first assessment, adolescents reported all previously mentioned control and study variables except for narcissism. One year apart, adolescents again completed those questionnaires and additionally reported their narcissistic traits. 7One year follow-up was chosen because autonomy-supportive parenting and prosocial behavior are relatively stable in the short run (Bülow et al., 2022; Te Brinke et al., 2023).

With the support of the principals of two public schools, the researchers recruited 650 adolescents who completed both assessments. Data from 21 adolescents were eliminated from the final sample due to substantial missing information relevant to the third study, resulting in a final sample of 629 adolescents. The average age of the sample was 12.86 years. Within the sample, 48.5% were girls. Participants’ family backgrounds can be viewed in Table S1.

### Measures

#### Prosocial Behavior

Prosocial behavior was assessed using the same brief scale applied in Study 1 because young adolescents might exhibit limited interest in participating in repeated measurements with long protocols, resulting in a high attrition rate and participation fatigue. Nevertheless, the researchers were fully aware that, when administrating the current investigation, additional strategies should be implemented since this scale exhibited a relatively low internal consistency in Study 1. The researchers thus used more age-appropriate verbal instructions and were more attentive to explaining each item when adolescents felt confused. At both time points, the internal consistency of this scale significantly improved in Study 3 compared to the first study (Time 1: Cronbach’s alpha = 0.70 and McDonald’s omega = 0.70; Time 2: Cronbach’s alpha = 0.75 and McDonald’s omega = 0.75).

#### Changes in Autonomy-Supportive Parenting

Autonomy-supportive parenting at both time points was assessed using the same instrument employed in Study 2. The scores of autonomy-supportive parenting, assessed at Time 2, were regressed on the same instrument measured at Time 1. The standardized residual scores were subsequently derived to represent the change in autonomy-supportive parenting between the two assessments. In this perspective, positive residual scores indicated an increased trend from Time 1 to Time 2, whereas negative scores represented the opposite trend. This calculation method was suggested by prior research (Huang et al., 2023) and offers the significant advantage of not inflating measurement errors. The internal consistency of this instrument in Study 3 was good at both Time 1 (Cronbach’s alpha = 0.87 and McDonald’s omega = 0.87) and Time 2 (Cronbach’s alpha = 0.94 and McDonald’s omega = 0.94).

#### Narcissism

Narcissism was assessed using the same scale used in Study 2. In Study 3, the internal consistency was adequate (Cronbach’s alpha = 0.70 and McDonald’s omega = 0.72).

#### Covariates

In Study 3, survey instructions (“all items administrated do not have right or wrong answers”) instead of validated scales were adopted to mitigate social desirability bias, given the extended duration of a repeated measurement study. In this scenario, reducing adolescents’ fatigue and participation burdens became the researchers’ priorities. It is important to note, however, that the longitudinal design implemented in Study 3 was instrumental in mitigating such concerns because consistent individual differences in prosocial behavior over time were controlled.

#### Missing Data and Attrition

Between the first and second waves of Study 3, there was an 18% attrition in the sample size, primarily attributed to student graduation or challenges in matching participants from the first time point. Of the data collected, 5% was missing, but these missing patterns were completely random. Independent *t*-tests comparing participants with and without missing data revealed no significant differences in key variables and covariates (*ts* < 1.80, *ps* > 0.07), with the exception of parental education level (*t* = −1.95, *p* = 0.05). Participants with lower parental education levels were more likely to drop out. However, this variable was adjusted for in all subsequent analyses. For the remaining missing data, a single imputation was employed, consistent with the technique employed in Studies 1 and 2.

#### Additional Analyses

Additional analyses in Study 3 were conducted to examine whether changes in teacher autonomy support played a similar role in controlling for the same covariates. This expansion would allow researchers to understand the contextual specificity in which narcissism played longitudinally (Belsky et al., 2022). In addition, changes in peer autonomy support were incorporated into the model, given the increasing peer interactions during adolescence (Brown & Larson, 2009). This incorporation was also relevant because prior research has shown that peer autonomy support interacting with dispositional traits to predict adolescents’ prosocial behavior (Ma et al., 2022). Finally, a post hoc power analysis was conducted.

## Results of Study 3

### Summary Statistics and Bivariate Correlations

**Fig. 6:**
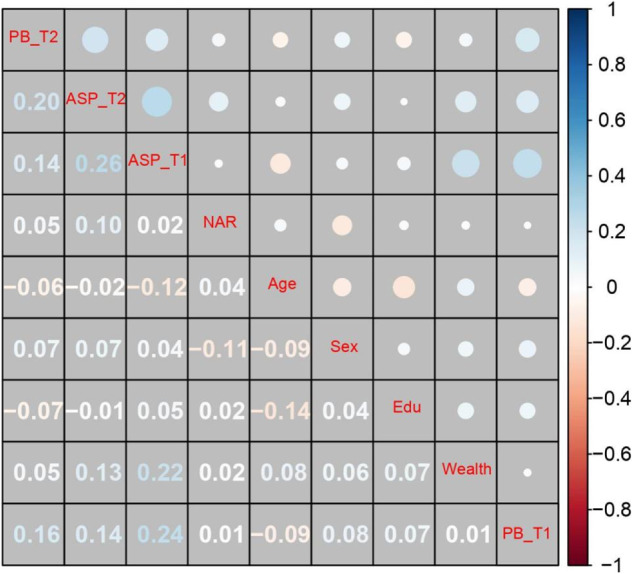
Correlation matrix in Study 3 (*N* = 629). Numbers and circles in blue font signify positive associations, while those in red indicate negative ones. More saturated colors and larger circles reflect stronger correlations. The descriptive statistics and the raw correlation matrix can be seen in Table S12. Sex was coded as 0 = girls and 1 = boys. PB prosocial behavior, ASP autonomy-supportive parenting, NAR narcissism, Edu parental education level, T1 Time 1, and T2 Time 2. ^*^p-value at a 0.05 level reached significance when *r* coefficients were more than 0.08

Summary statistics and the matrix of bivariate correlations between the study variables are presented in Table S13. As shown in Fig. 6, prosocial behavior (Time 2) was positively related to autonomy-supportive parenting assessed at both time points. Autonomy-supportive parenting and narcissism were only concurrently associated, and narcissism (Time 2) was not significantly related to prosocial behavior assessed at both time points.

### Hierarchical Regression Analysis Predicting Prosocial Behavior

Table 3 presents the regression analysis in Study 3. The overall model explained an 8% variance in prosocial behavior. In the second step, examination of the main effects accounted for an additional 3% variance. Change in autonomy-supportive parenting was positively related to prosocial behavior (Time 2). Beyond the main effects, a significant interaction effect between change in autonomy-supportive parenting and narcissism was found, explaining an additional 1% variance. Similar to those reported in Studies 1 and 2, the effect size of this interaction was small to medium. In Study 3, the first and second hypotheses were both supported.

**Table 3:** Hierarchical regression analysis predicting prosocial behavior in Study 3 (*N* = 629)

	*b*	*b SE*	95% CI for *b*		*β*	*t*	*p*	*R*^2^	△*R*^2^	△*F*
Step 1										
Age (Time 1)	−0.08	0.05	−0.18	0.02	−0.06	−1.58	0.11			
Sex (Time 1)^a^	0.20	0.17	−0.13	0.53	0.05	1.19	0.24			
Parental education (Time 1)	−0.30	0.12	−0.54	−0.05	−0.09	−2.38	0.02			
Family wealth (Time 1)	0.06	0.04	−0.03	0.14	0.05	1.32	0.19			
Prosocial behavior (Time 1)	0.15	0.04	0.08	0.22	0.16	4.06	<0.001	0.04	0.04	5.52^***^
Step 2										
Age (Time 1)	−0.08	0.05	−0.18	0.02	−0.07	−1.66	0.10			
Sex (Time 1)	0.18	0.17	−0.15	0.51	0.04	1.08	0.28			
Parental education (Time 1)	−0.28	0.12	−0.52	−0.04	−0.09	−2.30	0.02			
Family wealth (Time 1)	0.04	0.04	−0.04	0.13	0.04	1.06	0.29			
Prosocial behavior (Time 1)	0.14	0.04	0.07	0.21	0.15	3.78	<0.001			
ΔAutonomy-supportive parenting	0.31	0.08	0.14	0.47	0.14	3.66	<0.001			
Narcissism (Time 2)	0.16	0.17	−0.17	0.48	0.04	0.93	0.35	0.07	0.03	7.59^***^
Step 3										
Age (Time 1)	−0.08	0.05	−0.18	0.02	−0.06	−1.60	0.11			
Sex (Time 1)	0.20	0.17	−0.13	0.53	0.05	1.21	0.23			
Parental education (Time 1)	−0.27	0.12	−0.51	−0.03	−0.09	−2.23	0.03			
Family wealth (Time 1)	0.04	0.04	−0.05	0.12	0.03	0.85	0.40			
Prosocial behavior (Time 1)	0.14	0.04	0.07	0.21	0.15	3.86	<0.001			
ΔAutonomy-supportive parenting	−0.67	0.46	−1.57	0.22	0.15	−1.47	0.14			
Narcissism (Time 2)	0.14	0.17	−0.19	0.46	0.03	0.83	0.41			
ΔAutonomy-supportive parenting X Narcissism	0.34	0.16	0.03	0.65	0.08	2.18	0.03	0.08	0.01	4.74^*^

*p < 0.05, ***p < 0.001

^a^coded as 0 = girls and 1 = boys

**Fig. 7:**
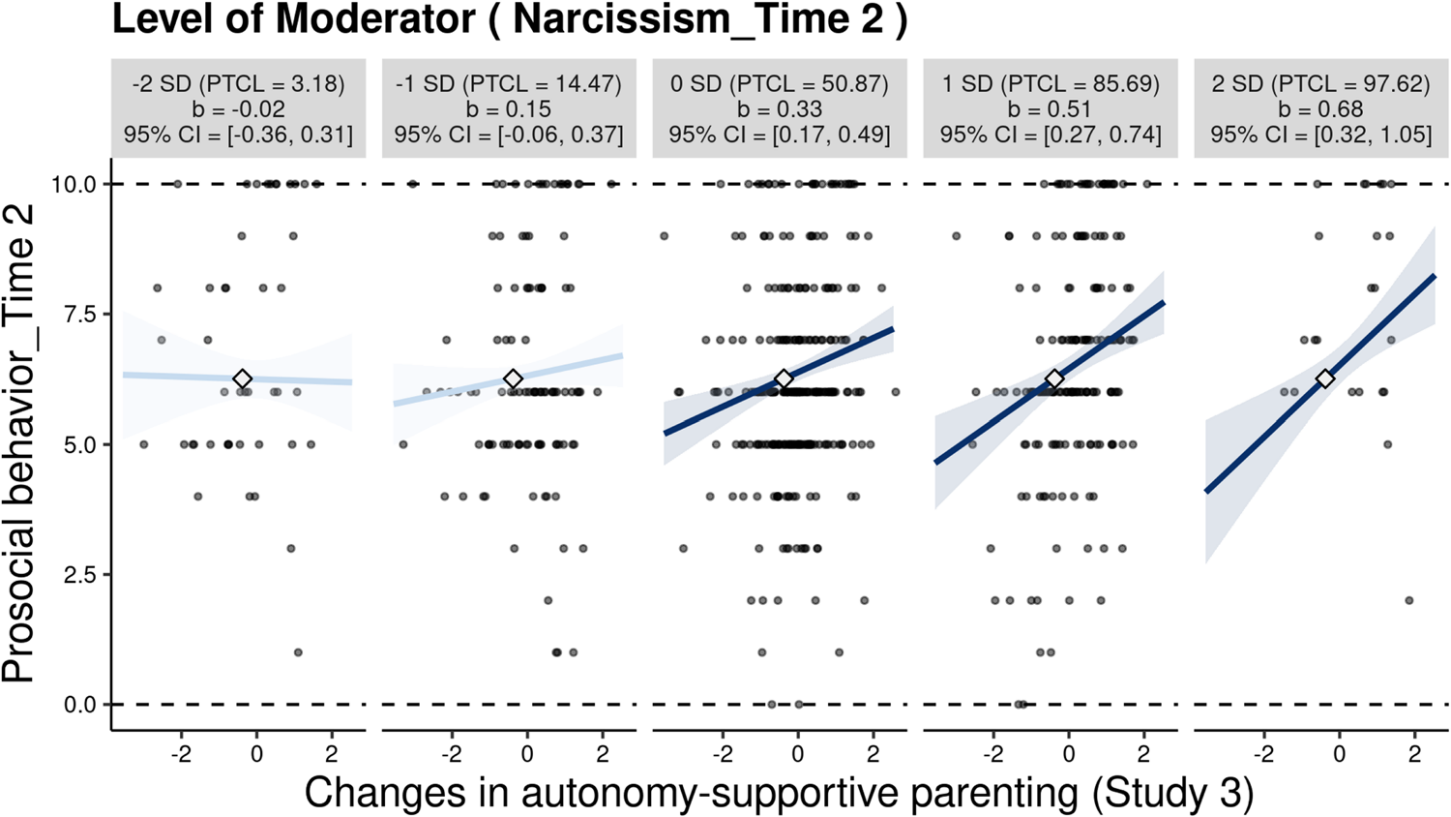
The moderating role of narcissism in the association between autonomy-supportive parenting and adolescents’ prosocial behavior in Study 3 (*N* = 629). Each graph displays the 95% confidence interval (CI) as a shaded area, the empirical data as gray circles, the maximum and minimum values of prosocial behavior as dashed horizontal lines, and the crossover point as a diamond. The x-axes represent the full range of changes in autonomy-supportive parenting (standardized residuals). PTCL percentile

The inspection of the two-way interaction exhibited similar patterns as those indicated in Studies 1 and 2 (see Fig. 7). Specifically, Study 3 revealed a positive correlation between changes in autonomy-supportive parenting and adolescents’ prosocial behavior at the mean level of narcissism, as well as at one and two standard deviations above this mean. However, this relationship was not significant at one and two standard deviations below the mean level of narcissism. As illustrated in Fig. S9, changes in autonomy-supportive parenting were positively associated with prosocial behavior in adolescents who scored −0.75 standard deviations above the mean in narcissism. Furthermore, 81.72% of the observations in narcissism were within this region of significance. In Study 3, the point estimate (*C* = −0.40, *SE* = 0.53) and the associated confidence boundaries (95% CI [−1.44, 0.64]) fell within the observed range of autonomy-supportive parenting. The differential susceptibility hypothesis of narcissism was again supported.

### Additional Analyses

First, as reported in Table S14, neither changes in teacher autonomy support nor changes in peer autonomy support exhibited significant main and interaction effects. Those supplementary analyses again suggested that the moderating role of narcissism was situationally specific to autonomy-supportive parenting. Second, a post hoc power analysis, indicated that the current sample size could yield approximately 95% statistical power (Sommet et al., 2023).

## Brief Discussion of Study 3

The findings from Study 3 complemented prior static and concurrent estimations of study associations, suggesting that change in autonomy-supportive parenting was positively related to prosocial behavior even after adjusting for the baseline level of prosocial behavior. In addition, narcissism moderated such a longitudinal process, for better *and* for worse. Despite establishing relatively consistent support for the interaction effect, the results of previous studies do not allow for causal inference. Study 4 aimed to fill in this gap by using a quasi-experimental design.

## Study 4

Using a randomized experiment, Study 4 adopted an autonomy-supportive parenting manipulation task and fortified the causal examination of the main and interactive associations with prosocial behavior.

## Methods of Study 4

### Participants and Procedure

With the permission of parents or legal guardians, adolescents were informed that they were signed up for an anonymous, voluntary, non-harm experimental study investigating memory recall and prosocial behavior. Data collection was conducted within a secondary school setting, with the permission and cooperation of the school administration. This study took place during regular school hours in designated classrooms, where the adolescents were first instructed to complete the manipulation task and then answer a few well-validated questionnaires. Upon completing the questionnaires, adolescents were allowed to enter a lottery, with the chance to receive 1–20 Chinese yuan as compensation.

Participants in Study 4 were 118 adolescents (*M*_age_ = 12.42; 53.4% girls) randomly assigned into two manipulation conditions: autonomy-supportive context (*n* = 59) and autonomy-suppressive context (*n* = 59). No significant differences in participants’ sociodemographic characteristics were revealed between the two conditions. Although obtaining a large and well-powered sample size is ideal for probing this hypothesized interaction effect, researchers must balance the resource limits and the desired statistical power, particularly considering that collecting quasi-experimental data at school become increasingly costly and challenging (Mayeux & Kraft, 2017). However, it should be noted that similarly sized adolescent samples have been employed in prior experimental studies (e.g., Li et al., 2023). Participants’ family backgrounds in Study 4 can be viewed in Table S1.

### Measures

Experimental study often operates under strict time constraints, necessitating more efficient data collection methods. Using the brief version of the scales allows researchers to focus on the core dimensions of the variable of interest and help prevent participant fatigue, ensuring that participants remain attentive and provide accurate responses throughout this investigation.

### Prosocial Behavior

Prosocial behavior was measured by a brief version of the scale employed in Study 2. This 15-item scale has been validated among Chinese adolescents, showing similar psychometric properties to the longer version (Zhang & Kou, 2011). In Study 4, the internal consistency improved compared to the second study (Cronbach’s alpha = 0.94 and McDonald’s omega = 0.94).

### Autonomy-Supportive Parenting Manipulation

Autonomy-supportive parenting was manipulated by instructing adolescents to vividly recall an experience related to interacting with their parents. This type of recall manipulation task has been demonstrated to be effective when studying social relationships (Dang & Liu, 2023). Specifically, adolescents were randomly assigned to one of the manipulation conditions (autonomy-supportive versus autonomy-suppressive). In the autonomy-supportive condition, adolescents were given written instructions, including several practices documented as autonomy-supportive interpersonal styles (e.g., acknowledging adolescents’ perspectives, providing choices, and meaningful rationales). In contrast, in the autonomy-suppressive condition, adolescents were given the meaningfully opposite instructions, containing controlling language by using verbs (e.g., “should”). Those instructions, described in detail in the supplementary materials, were crafted based on the conceptualization of autonomy support and prior research (Benita et al., 2014; Jungert et al., 2021). After reading those instructions, participants were first asked to recall relevant experiences and write down the real experiences in full detail and subsequently completed a four-item manipulation check questionnaire (e.g., “At this moment, I feel that my parents are willing to consider issues from my perspective.”; Cronbach’s alpha = 0.84 and McDonald’s omega = 0.84) rated on a 5-point scale.

### Narcissism

Narcissism was measured using the same scale employed in Study 2. In Study 4, the internal consistency was good (Cronbach’s alpha = 0.72 and McDonald’s omega = 0.74).

### Covariates

Due to the quantity constraints of this survey, an abridged five-item version of the social desirability scale (Schuessler et al., 1978) was administered in Study 4. The researchers intentionally limited this scale to the most representative items while including enough items to capture this construct effectively. The internal consistency of this scale was adequate (Cronbach’s alpha = 0.75 and McDonald’s omega = 0.74).

### Additional Analysis

The inherent challenge presented by a small sample size might increase the risk of generating biased model estimates. Monte Carlo cross-validation (with 200 repetitions and holding out 20% of the sample in each repetition) was thus conducted to provide a more robust and reliable estimate of model performance (Song et al., 2021). Specifically, Monte Carlo cross-validation randomly splits the data into training and validation sets over multiple iterations. This flexibility is particularly advantageous with a small data set, as it allows for a wide variety of data combinations, enhancing the robustness of the model estimate.

## Results of Study 4

### Manipulation Check

Adolescents in the autonomy-supportive condition reported significantly higher levels of autonomy-supportive parenting (*M* = 3.95, *SD* = 0.64) than those in the autonomy-suppressive condition (*M* = 2.60, *SD* = 0.36), *F* (1, 116) = 3.16, *p* < 0.001. The manipulation was therefore deemed effective in creating conditions that led to autonomous orientations.

### Summary Statistics and Bivariate Correlations

**Fig. 8:**
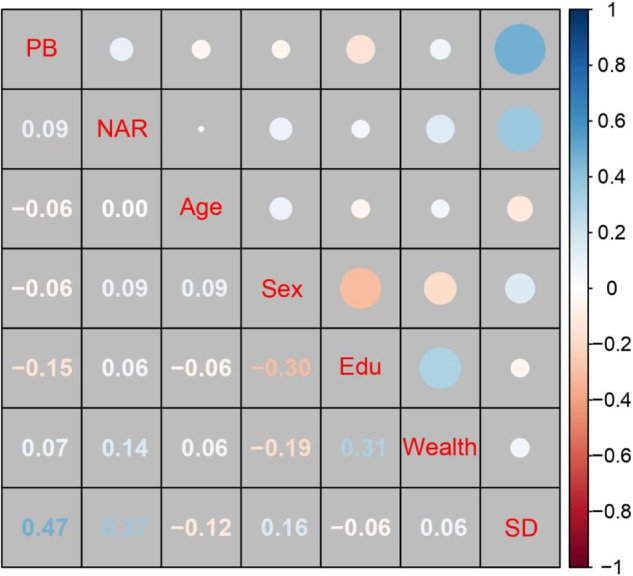
Correlation matrix in Study 3 (*N* = 629). Numbers and circles in blue font signify positive associations, while those in red indicate negative ones. More saturated colors and larger circles reflect stronger correlations. The descriptive statistics and the raw correlation matrix can be seen in Table S15. Sex was coded as 0 = girls and 1 = boys. PB prosocial behavior, NAR narcissism, Edu parental education level, and SD social desirability. ^*^p-value at a 0.05 level reached significance when *r* coefficients were more than 0.19

Summary statistics and intercorrelations for the study variables are provided in Table S15. Figure 8 visualizes the correlation matrix for the variables.

### Hierarchical Regression Analysis Predicting Prosocial Behavior

Table 4 summarizes the results of the regression analysis in Study 4. The mode totally explained a 36% variance in prosocial behavior. The main effects tested in the second step revealed no significant association between manipulation conditions and adolescents’ prosocial behavior. Yet the two-way interaction examined in the final step approached significant levels, additionally explaining up to a 7% variance. The effect size of this interaction was medium. The second hypothesis was again supported.

**Table 4:** Hierarchical regression analysis predicting prosocial behavior in Study 4 (*N* = 118)

	*b*	*b SE*	95% CI for *b*		*β*	*t*	*p*	*R*^2^	*ΔR*^2^	*ΔF*
Step 1										
Age	0.00	0.10	−0.19	0.19	0.00	−0.02	0.98			
Sex^a^	−0.33	0.16	−0.65	−0.01	−0.18	−2.07	0.04			
Parental education	−0.13	0.06	−0.25	−0.01	−0.19	−2.19	0.03			
Family wealth	0.05	0.06	−0.08	0.18	0.07	0.80	0.43			
Social desirability	0.45	0.08	0.30	0.60	0.48	5.83	<0.001	0.27	0.27	8.32^***^
Step 2										
Age	−0.03	0.10	−0.22	0.16	−0.03	−0.32	0.75			
Sex	−0.46	0.18	−0.82	−0.10	−0.25	−2.50	0.01			
Parental education	−0.15	0.06	−0.27	−0.03	−0.21	−2.40	0.02			
Family wealth	0.04	0.07	−0.09	0.17	0.06	0.65	0.51			
Social desirability	0.45	0.08	0.28	0.61	0.48	5.39	<0.001			
Manipulation conditions^b^	0.28	0.18	−0.08	0.64	0.30	1.53	0.13			
Narcissism	−0.15	0.16	−0.46	0.16	−0.08	−0.95	0.35	0.29	0.02	1.57
Step 3										
Age	0.04	0.10	−0.15	0.23	0.03	0.41	0.68			
Sex	−0.53	0.18	−0.88	−0.18	−0.29	−2.99	0.00			
Parental education	−0.14	0.06	−0.25	−0.02	−0.20	−2.39	0.02			
Family wealth	0.04	0.06	−0.08	0.17	0.06	0.70	0.49			
Social desirability	0.49	0.08	0.33	0.65	0.53	6.10	<0.001			
Manipulation conditions	−2.45	0.82	−4.07	−0.83	0.29	−3.00	0.00			
Narcissism	−0.69	0.22	−1.12	−0.25	−0.38	−3.15	0.00			
Manipulation conditions X Narcissism	1.00	0.29	0.42	1.59	0.55	3.42	<0.001	0.36	0.07	11.69^***^

***p < 0.001

^a^coded as 0 = girls and 1 = boys

^b^coded as 1 = autonomy-suppressive condition and 2 = autonomy-supportive condition

**Fig. 9:**
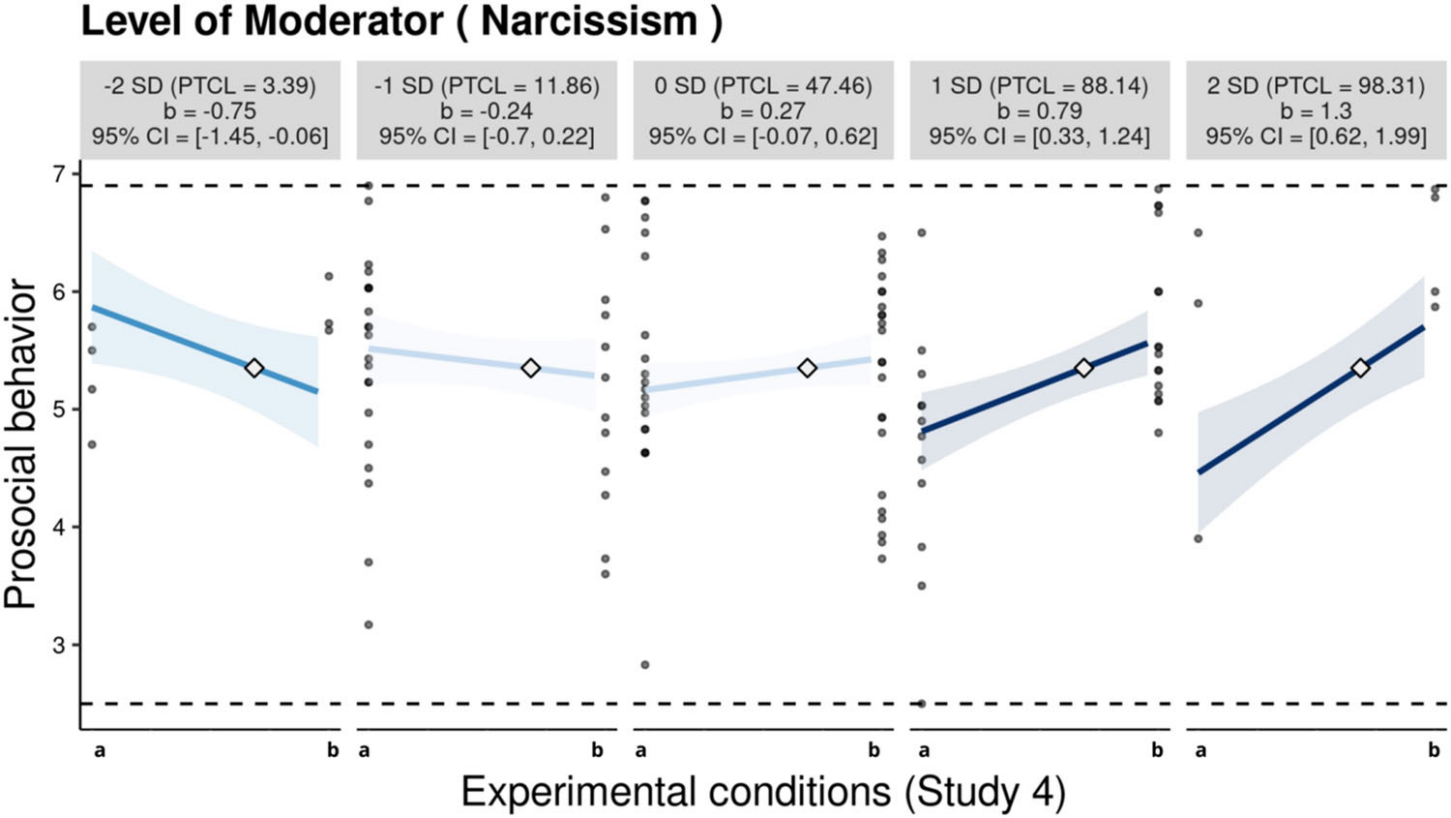
The moderating role of narcissism in the association between autonomy-supportive parenting and adolescents’ prosocial behavior in Study 4 (*N* = 118). Each graph displays the 95% confidence interval (CI) as a shaded area, the empirical data as gray circles, the maximum and minimum values of prosocial behavior as dashed horizontal lines, and the crossover point as a diamond. The x-axes represent two experimental conditions: (a) autonomy-suppressive condition and (b) autonomy-supportive condition. PTCL percentile

As shown in Fig. 9, at one and two standard deviations above the mean level of narcissism, adolescents in the autonomy-supportive condition exhibited significantly higher intercepts for prosocial behavior compared to those in the autonomy-suppressive condition. In contrast, at two standard deviations below the mean level of narcissism, adolescents in the autonomy-supportive condition displayed significantly lower intercepts for prosocial behavior than their counterparts in the suppressive condition. Furthermore, at the mean and one standard deviation from the mean level of narcissism, the intercept difference in prosocial behavior between the two conditions was not statistically significant. As depicted in Fig. S10, the experimental conditions showed a positive association with prosocial behavior in adolescents who scored 0.15 standard deviations above the mean in narcissism. Notably, 41.53% of the observations were within this region of significance.

The interaction patterns echoed those identified in the previous studies. Again, adolescents presenting high narcissism showed heightened susceptibility to both the beneficial and adverse effects of autonomy-supportive parenting. The inspection of point and interval estimates showed that the crossover point (*C* = 2.44, *SE* = 0.19) and 95% CI [2.06, 2.82] fell within the observed range of narcissism in Study 4.

### Additional Analysis

The results of cross-validation exhibited that when the regression model is generalized to another sample, 30% of the variance in prosocial behavior will likely be accounted for the variables under investigation. Additionally, the cross-validated mean square error of 0.68 suggested that, on average, the model predicting prosocial behavior scores will likely deviate from the observed scores in the new sample by 0.79 points on a 7-point scale. Given those prediction accuracy indices, the findings of Study 4 were validated internally and might have good generalization ability.

## Brief Discussion of Study 4

By employing a quasi-experimental design, Study 4 initially supported the causal interaction between autonomy-supportive parenting and narcissism on adolescents’ prosocial behavior. Adolescents scoring higher in narcissism seemed varied in the extent to which they were affected—for better *and* for worse—by autonomy-supportive parenting. Although this interaction has been cross-validated using the Monte Carlo procedure, the readers should interpret the current findings as “suggestive” instead of conclusive because probing the two-way interaction with a relatively limited sample size can still be challenging in the context of the confirmation of previous studies. A conclusive understanding would necessitate further empirical studies with significantly large sample sizes.

## Internal Meta-Analysis

Although the results were generally replicated in these four studies, two of them contained relatively limited sample sizes for estimating the two-way interaction. More pressingly, the shape of interaction patterns exhibited a slight disagreement, particularly in the context of low autonomy-supportive or narcissism. An internal meta-analysis was thus conducted to summarize the significance of the interaction and its effect size. The fixed effect mode was chosen in which each key effect size was weighted by sample size (Goh et al., 2016). Following the procedures outlined in prior research (Hasan-Aslih et al., 2019), *t*-values for the two-way interactions and associated simple slopes were first converted into Pearson’s *r* values and subsequently transformed using Fisher’s-*z* for the analyses. Overall, the interaction effect was significant, with a small-to-medium effect size (*Mr* = 0.12, 95% CI for *Mr* [0.08, 0.15], *Z* = 6.69, *p* < 0.001, two-tailed). A heterogeneity test across those four studies was not significant (*Q* within = 0.73, *p* = 0.87), indicating that the interaction under investigation might not be sample-specific.

Among adolescents scoring high in narcissism, the association between autonomy-supportive parenting and prosocial behavior was positively associated, with a medium-to-large effect size (*Mr* = 0.19 95% CI for *Mr* [0.15, 0.22], *Z* = 10.60, *p* < 0.001, two-tailed). In contrast, for those scoring low in narcissism, this association remained significantly positive, but the effect size was small (*Mr* = 0.04, 95% CI for *Mr* [0.01, 0.07], *Z* = 2.11, *p* = 0.04, two-tailed). Meta-analytically, the positive association between autonomy-supportive parenting and adolescents’ prosocial behavior was attenuated for adolescents reporting higher (versus lower) narcissism.

## General Discussion

Parenting that nurtures autonomy and the narcissistic traits of adolescents are pivotal in fostering youth prosocial behavior. However, the dynamics of how these two factors interactively influence such behavior in adolescents have yet to be fully elucidated. The current investigation aimed to bridge this knowledge gap by examining the main and interactive associations of autonomy-supportive parenting and narcissism with adolescents’ prosocial behavior. These aims were examined in a series of four studies that used different measures and research designs. Collectively, the findings from the four studies provide converging support, showing that high narcissism enhanced the positive relationship between autonomy-supportive parenting and adolescents’ prosocial behavior. The findings presented also constitute some of the first evidence considering narcissism as a differential susceptibility trait, although this hypothesis might be specific to certain dimensions of prosocial behavior. Below, the main findings and their theoretical and practical implications are discussed.

First, the current endeavor partially supported the first hypothesis, indicating a positive association between autonomy-supportive parenting and adolescents’ prosocial behavior. Such a finding aligns with past scholarship (Bülow et al., 2022; Nalipay et al., 2020) and corroborates mounting research conducted in East Asian societies (Lan et al., 2019; Ma et al., 2022). One possible interpretation for this positive association is grounded in the self-determination theory (Ryan & Deci, 2017, 2019), highlighting the fundamentally beneficial role of autonomy in optimal human functions. In the presence of high autonomy-supportive parenting, adolescents feel acknowledged and respected for their own perspectives, which might enhance feelings of subjective vitality and ultimately provide a situational foundation for the active engagement of prosocial behavior (Gagné, 2003). Another interpretation of this positive association aligns with prosocial behavior theory (Eisenberg et al., 2015). Parents who take adolescents’ perspectives and understand their mental states might facilitate the children’s perspective-taking and empathetic abilities, which are the core predictors of prosocial behavior. Nevertheless, notably, the main effect of autonomy-supportive parenting in Studies 1 and 4, which contained relatively smaller sample sizes, was not significant. This inconsistent main effect through the four studies may indicate that research estimates are unstable, particularly with regard to the studies with small sample sizes.

Second, this research suggested that adolescents’ narcissism moderated this positive association. The results from the four studies and an internal meta-analysis consistently showed that this positive association appeared to be more pronounced for those scoring higher (versus lower) narcissism. This observation aligns with emerging perspectives that a certain degree of self-focused attitudes, when balanced with autonomy-supportive situations, may foster social adeptness and proactive engagement in prosocial activities (Lan, 2023). Adaptive aspects of narcissism, such as healthy self-worth and resilience, can contribute positively to an adolescent’s interpersonal relationships and self-image (Miller et al., 2017; Xu et al., 2020). These attributes, when moderated and combined with autonomy-supportive parenting, may enhance adolescents’ prosocial behavior. Contrary to the consistent pattern observed in adolescents manifesting high narcissism, those scoring low in narcissism displayed varied interaction patterns across four studies. These discrepancies, as partially resolved by an internal meta-analysis, still supported a positive association for those scoring low in narcissism, but its strength was weak. Adolescents with low narcissism might already possess inherent qualities, such as empathy and cooperativeness. Hence, these adolescents may not require the same levels of autonomy support to exhibit prosocial behavior.

In addition, the crossover interaction patterns identified in Studies 1, 3, and 4 supported the differential susceptibility hypothesis of narcissism and corresponded with prior research (Lan, 2023). Such an interaction aligns with the differential susceptibility theory (Pluess, 2015), suggesting that adolescents with susceptible traits are sensitive to both the costs and benefits of parenting practices. Narcissistic adolescents, despite their outward confidence, often have fragile self-evaluations easily threatened by different situations, either supporting or thwarting adolescents’ autonomy needs (Bosson et al., 2003; Fernie et al., 2016). Adolescents manifesting high narcissism might be more susceptible than others to autonomy-supportive parenting due to regarding such situations as opportunities to seek validation and proclaim superiority (Lan, 2023). In the presence of high autonomy-supportive parenting, adolescents might feel that their sense of self-worth is bolstered and that their abilities are validated as parents acknowledge their perspectives; in this scenario, adolescents might be more likely than in other situations to engage in prosocial activities as a platform to showcase personal abilities and achievements. In contrast, in the presence of low autonomy-supportive parenting, adolescents might be preoccupied with opportunities to proclaim superiority (Bosson et al., 2003). Adolescents in such circumstances are not entirely convinced of self-worth and may perceive low autonomy-supportive parenting as threats to the adolescents’ superiority, heightening their fear of failure or negative judgment and compensating for their self-doubts by engaging less in prosocial activities.

However, this differential susceptibility hypothesis of narcissism was not fully supported in Study 2. When taking closer into different subscales of prosocial behavior, the results showed differential susceptibility for relational behavior but vantage sensitivity for the other three subscales of prosocial behavior. Several potential explanations might account for these differing patterns. Relational behavior, by definition, is more focused on social interactions and maintaining harmonious relationships (Yang et al., 2016). Adolescents scoring high in narcissism may be particularly sensitive to relational dynamics due to their heightened self-focus and desire for admiration. This could make them more responsive (positively or negatively) to the autonomy-supportive parenting they perceive, especially in contexts that affect their social standing or relationships. Hence, the differential susceptibility pattern might reflect the heightened sensitivity of narcissistic adolescents to relational cues and dynamics in their environment. This might be particularly acute because maintaining harmonious relationships and social interactions is important in collectivistic societies (Markus & Kitayama, 2010). Nevertheless, adolescents manifesting high narcissism might respond more positively to high autonomy-supportive parenting when it comes to altruistic behavior, behavior benefiting public welfare, and trait prosociality. This could be because these aspects of prosocial behavior are less directly linked to interpersonal dynamics and more related to general societal norms, self-image, and public welfare. High narcissism could enhance the association of high autonomy-supportive parenting with these behaviors, as these adolescents might use prosocial acts as a means to gain admiration, status, or self-worth.

### Limitations and Implications

Along with those findings, the current findings must also be evaluated within the context of several limitations. First, prosocial behavior in the present studies was predominantly constructed as a global and homogeneous variable. As indicated by the findings in Study 2, the interaction between autonomy-supportive parenting and narcissism might be distinctively linked to different types of prosocial behavior. Future research should develop a comprehensive evaluation of the prosocial behavior spectrum based on diverse motives, situations, and target to relate them to autonomy-supportive parenting and narcissism (Carlo & Padilla-Walker, 2020). Similarly, more recent theoretical movements have proposed differentiating several underlying components of narcissism (Crowe et al., 2019; Miller et al., 2021), although its multidimensional assessment in youth is still in infancy. Future research might therefore also consider unpacking each dimension of narcissism. Second, delving into the moderating role of narcissism holds significant theoretical and practical relevance, but the conditional process underlying the positive association between autonomy-supportive parenting and prosocial behavior is far more complex than what is investigated. One agenda for future initiatives should thus elaborate on this positive association by exploring additional dispositional moderators (e.g., sympathy; Xu & Zhang, 2023; grit; Lan et al., 2019) to discuss the unexplained variance found in the present studies. A third limitation that needs to be mentioned is that genetic factors might present a potential confound in the current findings because autonomy-supportive parents might transfer genetic dispositions associated with prosocial behavior to their children (Kretschmer, 2023). Future studies might use a genetically informed design to disentangle potential genetic and environmental processes that explain these study associations. Fourth, examining the crossover two-way interaction, by theory, requires a large sample size (Sommet et al., 2023), but unfortunately, due to time or financial constraints, two of the four studies contained limited sample sizes, which might generate biased estimates and inflated Type I errors. Thus, readers should be cautious when interpreting narcissism as a differential susceptibility trait, and future studies should replicate this finding using well-powered surveys/experiments with large samples. Finally, this research included only Chinese adolescents. Although that cultural context is well-suited to addressing study associations, one caveat is that the generalizability of the current findings might be restricted by certain cultural boundaries. Future studies should consider recruiting samples from multiple cultural contexts to replicate the present findings.

Those limitations notwithstanding, the present studies demonstrate important implications at both the theoretical and practical levels. Regarding theoretical implications, this research contributes to enriching the universality of the self-determination theory in an East Asian society. The findings also add to the growing bodies of work by adopting the comprehensive self-determination theory framework to gain a deep understanding of the complex interaction between socialization experiences and dispositional characteristics related to adolescents’ prosocial behavior. Further, exploring the association between narcissism and prosocial behavior contributes to discussing the general Dark Triad framework in relation to the association between narcissism and the remaining two Dark traits. The current findings challenge the predominantly negative connotation associated with narcissism, suggesting that its nuanced role in adolescents’ prosocial behavior warrants a more differentiated consideration. Especially under the influence of autonomy-supportive parenting, certain aspects of narcissism may paradoxically enhance adolescents’ prosocial behavior. Additionally, the present studies are of theoretical relevance to the differential susceptibility theory because narcissistic adolescents might exhibit pronounced responses to both negative and positive situational influences.

Through an examination of those associations with diverse research designs, this research also provides important insights into developing practical activities. First, the research suggests that facilitating autonomy-supportive parenting practices is beneficial to adolescents’ prosocial behavior. Educators and practitioners can, for instance, organize structured presentations via parent meetings online or at school, highlighting the critical roles of autonomy-supportive practices in adolescents’ prosocial behavior. During such meetings, educators and practitioners can also exemplify specific autonomy-supportive practices for parents, such as considering the adolescent’s point of view and providing meaningful rationales for their guidance or decisions. Importantly, the findings also indicate that autonomy-supportive parenting might not benefit all adolescents equally, arguing against the one-size-fits-all approach. The moderating role of narcissism played contributes to developing personalized initiatives that hold considerable promise for educators and practitioners. For instance, according to the assessment of autonomy-supportive parenting and narcissism, educators or practitioners can better locate adolescents in need of improved intervention or prevention efficacy.

## Conclusion

Autonomy-supportive parenting and narcissism are essential in terms of facilitating adolescents’ prosocial behavior. However, how these two factors interact and relate to adolescents’ prosocial behavior remains largely unexplored. The present investigation capitalizes on a series of four studies to extend previous scholarship by examining the main and interactive associations of autonomy-supportive parenting and narcissism with adolescents’ prosocial behavior. The findings converge to suggest that the positive association between autonomy-supportive parenting and adolescents’ prosocial behavior is amplified in the presence of high narcissism. The interaction pattern presented also suggests adolescents manifesting high narcissism exhibit heightened susceptibility to autonomy-supportive parenting than others, for better *and* for worse, although this pattern may be unique to certain aspects of prosocial behavior. Understanding how these factors interact is vital for propelling theoretical advancements and developing precise, tailored strategies to enhance prosocial behavior during adolescence.

### Supplementary Information

Supplementary Information

## Footnotes

1 One previous study has suggested that narcissism might be a potential mediator between autonomy-supportive parenting and Chinese adolescents’ developmental outcomes (Li et al., 2020). The present studies did not formulate this mediating role of narcissism in the tested hypothesis due primarily to the theoretical considerations. Methodologically, Studies 1 and 2 were based on cross-sectional designs, preventing the researchers from drawing valid conclusions regarding the causal mediation hypothesis (Maxwell & Cole, 2007). Although the longitudinal design of Study 3 might allow for such an analysis, preliminary correlation analysis showed no significant relationships between narcissism at Time 2 and prosocial behavior assessed at both time points. Therefore, such an analysis was not considered.

2 Power analysis calculation is one of the essential factors when designing the desired sample size, but the sample size that will be collected should not be mechanistically applied that statistics (Lakens, 2022). Notably, additional steps were taken to enhance each study’s statistical power, including employing well-validated scales with multiple items, controlling various covariates, and ensuring sample homogeneity (Sommet et al., 2023).

3 Each of the four studies achieved a response rate of over 90%, with minimal participant refusals. This outcome was unsurprising, given that the data collection was conducted through well-established school collaborations, where both school principals and teachers demonstrated high levels of cooperation and attentiveness to the research process. Although the researchers were unable to track the specific students who declined participation in the four studies, making it impossible to compare key variables between those who volunteered to participate and those who refused, the surveys with moderate-to-high response rates provided data that was assumed to be less biased (Vink et al., 2004).

4 Adjusting the significance threshold when conducting multiple studies/tests is a highly debated issue (Rubin, 2021). The present research chose not to adjust for alpha levels, avoiding putting a conservative threshold and potentially inhibiting novel discovery-oriented research, given that this research examined the rarely tested interaction on one single outcome. This non-adjustment also considered that each study was considered an independent analysis and that Studies 1 and 4 contained relatively limited sample sizes.

5 Prior meta-analytic findings have suggested no significant age differences in terms of the association between experiences of autonomy and adolescents’ prosocial behavior (Donald et al., 2021). Although the interaction between autonomy-supportive parenting and narcissism on adolescents’ prosocial behavior might be distinct since slightly different age groups of adolescents were recruited, analyses were controlled for adolescents’ age in all studies.

6 The present study did not utilize the widely employed multidimensional scale of the Prosocial Tendencies Measure (Carlo & Randall, 2002) because the findings from a previous validation study on mainland Chinese adolescents revealed a relatively ambiguous factorial structure (Kou et al., 2007). Additionally, specific subscales of this measure demonstrated notably low internal consistency (Kou et al., 2007). For a comprehensive discussion on the relationship between parenting and the multidimensional assessment of prosocial behavior in Chinese adolescents, readers might consult Ngai et al. (2018) and Xu & Zhang (2023).

7 Considering the stability of personality traits over time, narcissism was deliberately assessed only at Time 2. Therefore, maintaining participants from Time 1, despite their dropouts, and imputing their missing values at Time 2 for analyses was not possible. This was because, from the design perspective of Study 3, these specific data points were actually non-existent.

## References

[CR1] Belsky J, Zhang X, Sayler K (2022). Differential susceptibility 2.0: are the same children affected by different experiences and exposures?. Development and Psychopathology.

[CR2] Benita M, Roth G, Deci EL (2014). When are mastery goals more adaptive? It depends on experiences of autonomy support and autonomy. Journal of Educational Psychology.

[CR3] Bi X, Zhang L, Yang Y, Zhang W (2020). Parenting practices, family obligation, and adolescents‘ academic adjustment: cohort differences with social change in China. Journal of Research on Adolescence.

[CR4] Bosson JK, Brown RP, Zeigler-Hill V, Swann WB (2003). Self-enhancement tendencies among people with high explicit self-esteem: the moderating role of implicit self-esteem. Self and Identity.

[CR101] Boucher HC, Peng K, Shi J, Wang L (2009). Culture and implicit self-esteem: Chinese are “good” and “bad” at the same time. Journal of Cross-cultural Psychology.

[CR5] Boyce W, Torsheim T, Currie C, Zambon A (2006). The family affluence scale as a measure of national wealth: validation of an adolescent self-report measure. Social Indicators Research.

[CR6] Brown, B. B., & Larson, J. (2009). Peer relationships in adolescence. In R. M. Lerner, & L. Steinberg, (Eds.), *Handbook of adolescent psychology* (vol. 1, pp. 74-100). Hoboken, NJ: John Wiley & Sons, Inc.

[CR7] Brunell AB, Tumblin L, Buelow MT (2014). Narcissism and the motivation to engage in volunteerism. Current Psychology.

[CR8] Bülow A, Neubauer AB, Soenens B, Boele S, Denissen JJ, Keijsers L (2022). Universal ingredients to parenting teens: parental warmth and autonomy support promote adolescent well-being in most families. Scientific Reports.

[CR102] Cai H, Kwan VS, Sedikides C (2012). A Sociocultural Approach to Narcissism: The Case of Modern China. European Journal of Personality.

[CR9] Carlo G, Randall BA (2002). The development of a measure of prosocial behaviors for late adolescents. Journal of Youth and Adolescence.

[CR10] Carlo G, Padilla‐Walker L (2020). Adolescents ‘prosocial behaviors through a multidimensional and multicultural lens. Child Development Perspectives.

[CR12] Crowe ML, Lynam DR, Campbell WK, Miller JD (2019). Exploring the structure of narcissism: toward an integrated solution. Journal of Personality.

[CR13] Dang J, Liu L (2023). Do connectedness and satisfaction diminish or promote social goal-striving?. Personality and Social Psychology Bulletin.

[CR15] Derksen M, Morawski J (2022). Kinds of replication: examining the meanings of “conceptual replication” and “direct replication”. Perspectives on Psychological Science.

[CR16] Donald JN, Bradshaw EL, Conigrave JH, Parker PD, Byatt LL, Noetel M, Ryan RM (2021). Paths to the light and dark sides of human nature: a meta-analytic review of the prosocial benefits of autonomy and the antisocial costs of control. Psychological Bulletin.

[CR17] Eisenberg, N, Spinrad, T. L., & Knafo‐Noam, A. 2015). Prosocial development. In M. E. Lamb C. G. Coll (Eds.), *Handbook of child psychology and developmental science* (pp. 610–656). New York, NY: Wiley.

[CR18] Enders CK (2022). Applied missing data analysis 2.0.

[CR19] Faul F, Erdfelder E, Lang AG, Buchner A (2007). G* Power 3: a flexible statistical power analysis program for the social, behavioral, and biomedical sciences. Behavior Research Methods.

[CR20] Fernie BA, Fung A, Nikčević AV (2016). Different coping strategies amongst individuals with grandiose and vulnerable narcissistic traits. Journal of Affective Disorders.

[CR21] Gagné M (2003). The role of autonomy support and autonomy orientation in prosocial behavior engagement. Motivation and Emotion.

[CR22] Goh JX, Hall JA, Rosenthal R (2016). Mini meta-analysis of your own studies: some arguments on why and a primer on how. Social and Personality Psychology Compass.

[CR23] Gogol K, Brunner M, Goetz T, Martin R, Ugen S, Keller U, Preckel F (2014). “My questionnaire is too long!” The assessments of motivational-affective constructs with three-item and single-item measures. Contemporary Educational Psychology.

[CR24] Goodman SH, Lahey BB, Fielding B, Dulcan M, Narrow W, Regier D (1997). Representativeness of clinical samples of youths with mental disorders: a preliminary population-based study. Journal of Abnormal Psychology.

[CR25] Green SB (2010). How many subjects does it take to do a regression analysis. Multivariate Behavioral Research.

[CR26] Hasan-Aslih S, Pliskin R, van Zomeren M, Halperin E, Saguy T (2019). A darker side of hope: harmony-focused hope decreases collective action intentions among the disadvantaged. Personality and Social Psychology Bulletin.

[CR99] Harter, S. (2012). Emerging self-processes during childhood and adolescence. In M. R. Leary & J. P. Tangney (eds.), Handbook of self and identity (pp. 680–715). The Guilford Press.

[CR27] Huang J, Zhou L, Zhu D, Liu W, Lei J (2023). Changes in academic self-efficacy and value and crossover of burnout among adolescent students: a two-wave longitudinal study. Journal of Youth and Adolescence.

[CR28] Hui BP (2022). Prosocial behavior and well-being: shifting from the ‘chicken and egg to positive feedback loop. Current Opinion in Psychology.

[CR29] Javanbakht M, Lin J, Ragsdale A, Kim S, Siminski S, Gorbach P (2022). Comparing single and multiple imputation strategies for harmonizing substance use data across HIV-related cohort studies. BMC Medical Research Methodology.

[CR30] Jones DN, Paulhus DL (2014). Introducing the short dark triad (SD3), a brief measure of dark personality traits. Assessment.

[CR31] Jungert T, Schattke K, Proulx FA, Taylor G, Koestner R (2021). Whose autonomy support is more effective? Managers’ or co‐workers’? An experimental comparison of source and occupational context on intrinsic motivation. Canadian Journal of Administrative Sciences/Revue Canadienne des Sciences d’ l’Administration.

[CR32] Kauten RL, Barry CT (2016). Adolescent narcissism and its association with different indices of prosocial behavior. Journal of Research in Personality.

[CR33] Kauten R, Barry CT (2014). Do you think I’m as kind as I do? The relation of adolescent narcissism with self- and peer-perceptions of prosocial and aggressive behavior. Personality and Individual Differences.

[CR34] Kou Y, Hong HF, Tan C, Li L (2007). Revisioning prosocial tendencies measure for adolescents (in Chinese). Psychological Development and Education.

[CR35] Kretschmer T (2023). Parenting is genetically influenced: what does that mean for research into child and adolescent social development?. Social Development.

[CR36] Lakens D, Adolfi FG, Albers CJ, Anvari F, Apps MA, Argamon SE, Zwaan RA (2018). Justify your alpha. Nature Human Behaviour.

[CR37] Lakens D (2022). Sample size justification. Collabra Psychology.

[CR96] Lan X, Marci T, Moscardino U (2019). Parental autonomy support, grit, and psychological adjustment in Chinese adolescents from divorced families. Journal of Family Psychology.

[CR97] Lan, X., & Wang, W. (2020). Is early left-behind experience harmful to prosocial behavior of emerging adult? The role of parental autonomy support and mindfulness. *Current Psychology*, 1–14. 10.1007/s12144-020-00706-3.

[CR38] Lan X (2021). Disengaged and highly harsh? Perceived parenting profiles, narcissism, and loneliness among adolescents from divorced families. Personality and Individual Differences.

[CR39] Lan, X. (2023). Left-behind youth are not always bad! Relations between teacher autonomy support, narcissism, and prosocial behavior. *Current Psychology*, 1–11. 10.1007/s12144-022-03610-0.

[CR40] Long J.A. (2022). *interactions: Comprehensive, User-Friendly Toolkit for Probing Interactions*. R package version 1.1.6, https://cran.r-project.org/package=interactions.

[CR41] Li R, Yao M, Chen Y, Liu H (2020). Parent autonomy support and psychological control, dark triad, and subjective well-being of Chinese adolescents: synergy of variable-and person-centered approaches. The Journal of Early Adolescence.

[CR42] Li X, Ang RP (2019). Parental arrest and adolescent delinquency in Singapore: the moderating roles of narcissism, callous-unemotional traits, and impulsivity. Journal of Child and Family Studies.

[CR43] Li X, Li Z, Jiang J, Yan N (2023). Child’en’s sensory processing sensitivity and prosocial behaviors: testing the differential susceptibility theory. Journal of Experimental Psychology General.

[CR44] Little RJ (1988). A test of missing completely at random for multivariate data with missing values. Journal of the American Statistical Association.

[CR45] Lin H (2020). Probing two-way moderation effects: a review of software to easily plot Johnson-Neyman figures. Structural Equation Modeling: A Multidisciplinary Journal.

[CR46] Liu SK, Chien YL, Shang CY, Lin CH, Liu YC, Gau SSF (2013). Psychometric properties of the Chinese version of the Strength and Difficulties Questionnaire. Comprehensive Psychiatry.

[CR47] Ma C, Mastrotheodoros S, Lan X (2022). Linking classmate autonomy support with prosocial behavior in Chinese left-behind adolescents: the moderating role of self-esteem and grit. Personality and Individual Differences.

[CR48] Mageau GA, Ranger F, Joussemet M, Koestner R, Moreau E, Forest J (2015). Validation of the perceived parental autonomy support scale (P-PASS). Canadian Journal of Behavioural Science/Revanadiennenne des sciences du comportement.

[CR49] Malti T, Dys SP (2018). From being nice to being kind: development of prosocial behaviors. Current Opinion in Psychology.

[CR50] Markus HR, Kitayama S (2010). Cultures and selves: a cycle of mutual constitution. Perspectives on Psychological Science.

[CR51] Mayeux L, Kraft C (2017). Logistical challenges and opportunities for conducting peer nomination research in schools. In Peter E. L. Marks & Antonius H. N. Cillessen (Eds.), New Directions in Peer Nomination Methodology. New Directions for Child and Adolescent Development.

[CR52] Maxwell SE, Cole DA (2007). Bias in cross-sectional analyses of longitudinal mediation. Psychological Methods.

[CR53] McCabe C, Kim D, King K (2018). Improving present practices in the visual display of interactions. Advances in Methods and Practices in Psychological Science.

[CR54] McCurdy AL, Williams KN, Lee GY, Benito‐Gomez M, Fletcher AC (2020). Measurement of parental autonomy support: a review of theoretical concerns and developmental considerations. Journal of Family Theory & Review.

[CR55] Miller JD, Lynam DR, Hyatt CS, Campbell WK (2017). Controversies in narcissism. Annual Review of Clinical Psychology.

[CR56] Miller JD, Back MD, Lynam DR, Wright AG (2021). Narcissism today: what we know and what we need to learn. Current Directions in Psychological Science.

[CR57] Nalipay MJN, King RB, Cai Y (2020). Autonomy is equally important across East and West: testing the cross-cultural universality of self-determination theory. Journal of Adolescence.

[CR58] Ngai SSY, Xie L, Ng YH, Ngai HL (2018). The effects of parenting behavior on prosocial behavior of Chinese adolescents in Hong Kong. Children and Youth Services Review.

[CR100] Nielsen M, Haun D, Kärtner J, Legare CH (2017). The persistent sampling bias in developmental psychology: A call to action. Journal of Experimental Child Psychology.

[CR59] Ouyang M, Cai X, Yin Y, Zeng P, Chen Y, Wang X, Wang P (2020). Student-student relationship and adolescent problematic smartphone use: the mediating role of materialism and the moderating role of narcissism. Children and Youth Services Review.

[CR98] Paulhus DL, Williams KM (2002). The dark triad of personality: Narcissism, Machiavellianism, and psychopathy. Journal of Research in Personality.

[CR61] Pfattheicher S, Nielsen YA, Thielmann I (2022). Prosocial behavior and altruism: a review of concepts and definitions. Current Opinion in Psychology.

[CR62] Pluess M, Belsky J (2013). Vantage sensitivity: individual differences in response to positive experiences. Psychological Bulletin.

[CR63] Pluess M (2015). Individual differences in environmental sensitivity. Child Development Perspectives.

[CR64] R Core Team. (2022). R: A language and environment for statistical computing.

[CR65] Rohrer JM, Arslan RC (2021). Precise answers to vague questions: Issues with interactions. Advances in Methods and Practices in Psychological Science.

[CR66] Ryan, R. M., & Deci, E. L. (2017). *Self-determination theory: basic psychological needs in motivation, development, and wellness*. Guilford Publications.

[CR67] Ryan, R. M., & Deci, E. L. (2019). *Brick by brick: the origins, development, and future of self-determination theory*. In Advances in motivation science (Vol. 6, pp. 111-156). Elsevier.

[CR68] Rubin DB (1987). Multiple imputation for nonresponse in surveys.

[CR69] Rubin M (2021). When to adjust alpha during multiple testing: a consideration of disjunction, conjunction, and individual testing. Synthese.

[CR70] Schuessler K, Hittle D, Cardascia J (1978). Measuring responding desirably with attitude-opinion items. Social Psychology.

[CR71] Sommet N, Weissman DL, Cheutin N, Elliot AJ (2023). How many participants do I need to test an interaction? Conducting an appropriate power analysis and achieving sufficient power to detect an interaction. Advances in Methods and Practices in Psychological Science.

[CR72] Song QC, Tang C, Wee S (2021). Making sense of model generalizability: a tutorial on cross-validation in R and Shiny. Advances in Methods and Practices in Psychological Science.

[CR73] Streit, C., McGinley, M., & Carlo, G. (2023). A systemic, multiple socialization approach to the study of prosocial development. *Journal of Social and Personal Relationships*, 02654075231196595. 10.1177/02654075231196595

[CR74] Te Brinke LW, van de Groep S, van Der Cruijsen R, Crone EA (2023). Variability and change in adolescents’ prosocial behavior across multiple time scales. Journal of Research on Adolescence.

[CR75] Teuber, Z., Schreiber, S., Rueth, J. E., & Lohaus, A. (2022). Emotion regulation among Chinese and German children and adolescents: a binational comparative study. *Current Psychology*, 1–15. 10.1007/s12144-022-03578-x.

[CR76] Thielmann I, Spadaro G, Balliet D (2020). Personality and prosocial behavior: a theoretical framework and meta-analysis. Psychological Bulletin.

[CR77] Thomaes S, Stegge H, Bushman BJ, Olthof T, Denissen J (2008). Development and validation of the Childhood Narcissism Scale. Journal of Personality Assessment.

[CR78] Truhan, T. E., Sedikides, C., Armour, C., & Papageorgiou, K. A. (2023). A tri-directional examination of adolescent personality, perceived parenting, and economic and parental adversity contexts in influencing adolescent behavioral outcomes. *Journal of Adolescence*. 10.1002/jad.12223. 10.1002/jad.12223 37504510

[CR80] Vasquez AC, Patall EA, Fong CJ, Corrigan AS, Pine L (2016). Parent autonomy support, academic achievement, and psychosocial functioning: a meta-analysis of research. Educational Psychology Review.

[CR81] Vink JM, Willemsen G, Stubbe JH, Middeldorp CM, Ligthart RS, Baas KD, Boomsma DI (2004). Estimating non-response bias in family studies: application to mental health and lifestyle. European Journal of Epidemiology.

[CR82] Vrolijk P, Van Lissa CJ, Branje SJ, Meeus WH, Keizer R (2020). Longitudinal linkages between father and mother autonomy support and adolescent problem behaviors: between-family differences and within-family effects. Journal of Youth and Adolescence.

[CR83] Wang Q, Pomerantz EM, Chen H (2007). The role of parents’ control in early adolescents’ psychological functioning: a longitudinal investigation in the United States and China. Child Development.

[CR84] Wei, T., & Simko, V. (2021). R package “corrplot”: visualization of a correlation matrix (Version 0.84). https://github.com/taiyun/corrplot.

[CR85] Widaman KF, Helm JL, Castro-Schilo L, Pluess M, Stallings MC, Belsky J (2012). Distinguishing ordinal and disordinal interactions. Psychological Methods.

[CR87] Xu X, Huebner ES, Tian L (2020). Profiles of narcissism and self-esteem associated with comprehensive mental health in adolescents. Journal of Adolescence.

[CR88] Xu Y, Hamamura T (2014). Folk beliefs of cultural changes in China. Frontiers in Psychology.

[CR89] Xu J, Zhang H (2023). Parenting and Chinese adolescents’ multidimensional prosocial behaviors: the moderating role of sympathy. The Journal of Psychology.

[CR90] Yang Y, Zhang M, Kou Y (2016). The revalidation and development of the prosocial behavior scale for adolescent (in Chinese). Chinese Social Psychology Review.

[CR91] Zeng R, Greenfield PM (2015). Cultural evolution over the last 40 years in China: using the Google Ngram Viewer to study implications of social and political change for cultural values. International Journal of Psychology.

[CR92] Zhang J, Ziegler M, Paulhus DL (2019). Development and evaluation of the Short Dark Triad-Chinese version (SD3-C). Current Psychology.

[CR93] Zhang Q, Kou Y (2011). The dimension of measurement on prosocial behavior: exploration and confirmation (in Chinese). Sociological Studies.

[CR94] Zheng Y, McMahon RJ (2022). Lability in parental warmth in childhood: antecedents and early adolescent outcomes. Journal of Clinical Child & Adolescent Psychology.

[CR95] Zhou Z, Qu Y, Li X (2022). Parental collectivism goals and Chinese adolescents’ prosocial behaviors: the mediating role of authoritative parenting. Journal of Youth and Adolescence.

