# Supplementary material for: Narcissism Moderates the Association Between Autonomy-Supportive Parenting and Adolescents’ Prosocial Behavior
Source: J Youth Adolesc. 2023 Dec 26;53(3):632–55. doi: 10.1007/s10964-023-01933-0 (PMC10838263; doi:10.1007/s10964-023-01933-0)

**Lan, X., & Ma, C. (2023). Narcissism Moderates the Association Between Autonomy-Supportive Parenting and Adolescents’ Prosocial Behavior**

**Table S1.** *Basic socio-demographic characteristics of the samples in Studies 1-4.*

| Variables | Study 1  (*N* = 318) | Study 2  (*N* = 2,098) | Study 3  (*N* = 629) | Study 4  (*N* = 118) |
| --- | --- | --- | --- | --- |
| **Age (years)** | 12.91 ± 1.63 | 15.70 ± 1.55 | 12.86 ± 1.70 | 12.42 ± 0.80 |
| **Sex** |  |  |  |  |
| Girls | 167 (52.5%) | 925 (44.1%) | 305 (48.5%) | 63 (53.4%) |
| Boys | 151 (47.5%) | 1173 (55.9%) | 324 (51.5%) | 55 (46.6%) |
| **Parental education (mothers/fathers)** |  |  |  |  |
| Middle school or lower | 118 (37.1%)/135 (42.5%) | 623 (29.7%)/585 (27.9%) | 332 (52.8%)/373 (59.3%) | 77 (65.3%)/62 (52.5%) |
| High school | 147 (46.2%)/110 (34.5%) | 1134 (54.1%)/1138 (54.2%) | 284 (45.1%)/227 (36.1%) | 24 (20.3%)/36 (30.5%) |
| University degree or higher | 53 (16.7%)/73 (23.0%) | 341 (16.3%)/375 (17.9%) | 13 (2.1%)/29 (4.6%) | 17 (14.4%)/20 (17.0%) |
| **Family wealth ^a^** |  |  |  |  |
| Low income | 66 (20.8%) | 679 (32.4%) | 182 (28.9%) | 44 (37.3%) |
| Medium income | 145 (45.6%) | 1012 (48.2%) | 305 (48.5%) | 59 (50.0%) |
| High income | 107 (33.6%) | 407 (19.4%) | 142 (22.6%) | 15 (12.7%) |

*Note*. ^a^ The sociodemographic information of Study 3 was derived from the data collected at the first time point, and the Family Affluence Scale in Studies 1 and 3 was used as a proxy representing adolescents’ family wealth.

**Study 1**

**Table S2**. *Descriptive statistics, correlations, and inter-item reliabilities for scales used in Study 1* (*N* = 318)

| Variables | 1 | 2 | 3 | 4 | 5 | 6 | 7 |
| --- | --- | --- | --- | --- | --- | --- | --- |
| 1. Prosocial behavior | — |  |  |  |  |  |  |
| 2. Autonomy-supportive parenting | 0.14^*^ | — |  |  |  |  |  |
| 3. Narcissism | 0.16^**^ | 0.31^***^ | — |  |  |  |  |
| 4.Age | -0.09 | -0.09 | 0.05 | — |  |  |  |
| 5. Sex ^a^ | 0.00 | 0.06 | 0.11^*^ | 0.01 | — |  |  |
| 6. Parental education | 0.06 | -0.01 | 0.01 | -0.45^***^ | 0.13^*^ | — |  |
| 7. Family wealth | 0.17^**^ | 0.12^*^ | 0.06 | -0.36^***^ | -0.01 | 0.26^***^ | — |
| Mean | 7.14 | 3.58 | 2.48 | 12.91 | — | 3.62 | 4.42 |
| *SD* | 1.96 | 0.86 | 0.53 | 1.63 | — | 1.22 | 2.10 |
| Minimum | 1.00 | 1.00 | 1.00 | 10.00 | 0.00 | 2.00 | 0.00 |
| Maximum | 10.00 | 5.00 | 4.00 | 15.00 | 1.00 | 6.00 | 9.00 |
| Skewness | -0.39 | -0.72 | 0.14 | -0.29 | — | 0.42 | -0.25 |
| Kurtosis | -0.45 | 0.44 | 0.32 | -1.15 | — | -0.46 | -0.55 |
| α/ϖ | 0.65/0.65 | 0.89/0.89 | 0.79/0.79 | — | — | — | — |

*Note*. ^a^ coded as 0 = girls and 1 = boys.

^*^ *p* < .05, ^**^ *p* < .01, ^***^ *p* < .001.

**Table S3.** *Hierarchical regression analysis predicting prosocial behavior with the scores of father autonomy support in Study 1* (*N* = 318)

|  | *b* | *b SE* | 95% CI for *b* | | *β* | *t* | *p* | *R*^2^ | △*R*^2^ | | △*F* |
| --- | --- | --- | --- | --- | --- | --- | --- | --- | --- | --- | --- |
| **Step 1** |  |  |  |  |  |  |  |  | |  |  |
| Age | -0.04 | 0.08 | -0.19 | 0.12 | -0.03 | -0.45 | 0.65 |  | |  |  |
| Sex ^a^ | -0.01 | 0.22 | -0.44 | 0.43 | 0.00 | -0.03 | 0.98 |  | |  |  |
| Parental education | 0.01 | 0.10 | -0.19 | 0.21 | 0.00 | 0.07 | 0.94 |  | |  |  |
| Family wealth | 0.15 | 0.06 | 0.04 | 0.26 | 0.16 | 2.67 | 0.01 | 0.03 | | 0.03 | 2.47^*^ |
| **Step 2** |  |  |  |  |  |  |  |  | |  |  |
| Age | -0.04 | 0.08 | -0.20 | 0.11 | -0.04 | -0.55 | 0.58 |  | |  |  |
| Sex | -0.10 | 0.22 | -0.52 | 0.33 | -0.05 | -0.43 | 0.66 |  | |  |  |
| Parental education | 0.01 | 0.10 | -0.18 | 0.21 | 0.01 | 0.15 | 0.88 |  | |  |  |
| Family wealth | 0.13 | 0.06 | 0.02 | 0.24 | 0.13 | 2.25 | 0.03 |  | |  |  |
| Father autonomy support | 0.23 | 0.13 | -0.02 | 0.48 | 0.10 | 1.78 | 0.08 |  | |  |  |
| Narcissism | 0.48 | 0.21 | 0.06 | 0.90 | 0.13 | 2.25 | 0.03 | 0.06 | | 0.03 | 5.71^**^ |
| **Step 3** |  |  |  |  |  |  |  |  | |  |  |
| Age | -0.05 | 0.08 | -0.20 | 0.10 | -0.04 | -0.70 | 0.49 |  | |  |  |
| Sex | -0.03 | 0.22 | -0.46 | 0.39 | -0.02 | -0.16 | 0.87 |  | |  |  |
| Parental education | -0.02 | 0.10 | -0.22 | 0.17 | -0.01 | -0.23 | 0.82 |  | |  |  |
| Family wealth | 0.14 | 0.06 | 0.03 | 0.25 | 0.15 | 2.50 | 0.01 |  | |  |  |
| Father autonomy support | -1.15 | 0.51 | -2.15 | -0.16 | 0.13 | -2.28 | 0.02 |  | |  |  |
| Narcissism | -1.62 | 0.77 | -3.14 | -0.10 | 0.13 | -2.09 | 0.04 |  | |  |  |
| Father autonomy support X Narcissism | 0.58 | 0.21 | 0.18 | 0.99 | 0.14 | 2.82 | 0.01 | 0.09 | | 0.03 | 7.95^**^ |

*Note*. ^a^ coded as 0 = girls and 1 = boys.

^*^ *p* < .05, ^**^ *p* < .01.

**Table S4.** *Hierarchical regression analysis predicting prosocial behavior with the scores of mother autonomy support in Study 1* (*N* = 318)

|  | *b* | *b SE* | 95% CI for *b* | | *β* | *t* | *p* | *R*^2^ | △*R*^2^ | | △*F* |
| --- | --- | --- | --- | --- | --- | --- | --- | --- | --- | --- | --- |
| **Step 1** |  |  |  |  |  |  |  |  | |  |  |
| Age | -0.04 | 0.08 | -0.19 | 0.12 | -0.03 | -0.45 | 0.65 |  | |  |  |
| Sex ^a^ | -0.01 | 0.22 | -0.44 | 0.43 | 0.00 | -0.03 | 0.98 |  | |  |  |
| Parental education | 0.01 | 0.10 | -0.19 | 0.21 | 0.00 | 0.07 | 0.94 |  | |  |  |
| Family wealth | 0.15 | 0.06 | 0.04 | 0.26 | 0.16 | 2.67 | 0.01 | 0.03 | | 0.03 | 2.47^*^ |
| **Step 2** |  |  |  |  |  |  |  |  | |  |  |
| Age | -0.04 | 0.08 | -0.20 | 0.11 | -0.03 | -0.52 | 0.60 |  | |  |  |
| Sex | -0.08 | 0.22 | -0.51 | 0.35 | -0.04 | -0.38 | 0.71 |  | |  |  |
| Parental education | 0.02 | 0.10 | -0.18 | 0.21 | 0.01 | 0.15 | 0.88 |  | |  |  |
| Family wealth | 0.14 | 0.06 | 0.03 | 0.25 | 0.15 | 2.43 | 0.02 |  | |  |  |
| Autonomy-supportive parenting | 0.10 | 0.12 | -0.15 | 0.34 | 0.05 | 0.78 | 0.43 |  | |  |  |
| Narcissism | 0.54 | 0.22 | 0.11 | 0.96 | 0.15 | 2.49 | 0.01 | 0.06 | | 0.03 | 4.40^**^ |
| **Step 3** |  |  |  |  |  |  |  |  | |  |  |
| Age | -0.05 | 0.08 | -0.20 | 0.10 | -0.04 | -0.63 | 0.53 |  | |  |  |
| Sex | -0.05 | 0.22 | -0.48 | 0.38 | -0.02 | -0.21 | 0.83 |  | |  |  |
| Parental education | -0.02 | 0.10 | -0.22 | 0.18 | -0.01 | -0.15 | 0.88 |  | |  |  |
| Family wealth | 0.15 | 0.06 | 0.04 | 0.26 | 0.16 | 2.66 | 0.01 |  | |  |  |
| Autonomy-supportive parenting | -1.05 | 0.48 | -1.99 | -0.11 | 0.07 | -2.20 | 0.03 |  | |  |  |
| Narcissism | -1.19 | 0.73 | -2.62 | 0.24 | 0.15 | -1.63 | 0.10 |  | |  |  |
| Autonomy-supportive parenting X Narcissism | 0.48 | 0.19 | 0.10 | 0.87 | 0.12 | 2.48 | 0.01 | 0.08 | | 0.02 | 6.16^**^ |

*Note*. ^a^ coded as 0 = girls and 1 = boys.

^*^ *p* < .05, ^**^ *p* < .01.

**Table S5.** *Poisson regression analysis predicting prosocial behavior in Study 1* (*N* = 318)

|  | *b* | *b SE* | 95% CI for *b* | | *β* | *t* | *p* | *Pseudo R*^2^ | *△R^2^* | | χ^2^ |
| --- | --- | --- | --- | --- | --- | --- | --- | --- | --- | --- | --- |
| **Step 1** |  |  |  |  |  |  |  |  | |  |  |
| Age | -0.01 | 0.02 | -0.03 | 0.02 | 0.99 | -0.34 | 0.74 |  | |  |  |
| Sex ^a^ | 0.00 | 0.04 | -0.08 | 0.08 | 1.00 | -0.03 | 0.97 |  | |  |  |
| Parental education | 0.00 | 0.02 | -0.04 | 0.04 | 1.00 | 0.06 | 0.95 |  | |  |  |
| Family wealth | 0.02 | 0.01 | 0.00 | 0.04 | 1.02 | 1.93 | 0.05 | 0.01 | | 0.01 | - |
| **Step 2** |  |  |  |  |  |  |  |  | |  |  |
| Age | -0.01 | 0.02 | -0.04 | 0.02 | 0.99 | -0.37 | 0.71 |  | |  |  |
| Sex | -0.01 | 0.04 | -0.10 | 0.07 | 0.99 | -0.29 | 0.77 |  | |  |  |
| Parental education | 0.00 | 0.02 | -0.04 | 0.04 | 1.00 | 0.11 | 0.91 |  | |  |  |
| Family wealth | 0.02 | 0.01 | 0.00 | 0.04 | 1.02 | 1.68 | 0.09 |  | |  |  |
| Autonomy-supportive parenting | 0.03 | 0.03 | -0.02 | 0.08 | 1.03 | 1.01 | 0.31 |  | |  |  |
| Narcissism | 0.07 | 0.04 | -0.01 | 0.15 | 1.07 | 1.67 | 0.10 | 0.03 | | 0.02 | 11.54^**^ |
| **Step 3** |  |  |  |  |  |  |  |  | |  |  |
| Age | -0.01 | 0.02 | -0.04 | 0.02 | 0.99 | -0.45 | 0.66 |  | |  |  |
| Sex | -0.01 | 0.04 | -0.09 | 0.08 | 0.99 | -0.12 | 0.90 |  | |  |  |
| Parental education | 0.00 | 0.02 | -0.04 | 0.04 | 1.00 | -0.16 | 0.88 |  | |  |  |
| Family wealth | 0.02 | 0.01 | 0.00 | 0.04 | 1.02 | 1.84 | 0.07 |  | |  |  |
| Autonomy-supportive parenting | 0.04 | 0.03 | -0.02 | 0.09 | 1.04 | 1.33 | 0.18 |  | |  |  |
| Narcissism | 0.07 | 0.04 | -0.02 | 0.15 | 1.07 | 1.57 | 0.12 |  | |  |  |
| Autonomy-supportive parenting X Narcissism | 0.08 | 0.04 | 0.00 | 0.16 | 1.08 | 1.97 | 0.04 | 0.05 | | 0.02 | 8.64^**^ |

*Note*. ^a^ coded as 0 = girls and 1 = boys.

^**^ *p* < .01.

**Table S6.** *Hierarchical regression analysis predicting prosocial behavior in Study 1 with regression-based multiple imputations* (*N =* 318)

|  | *b* | *b SE* | 95% CI for *b* | | *t* | *p* | *R*^2^ | △*R*^2^ | | △*F* |
| --- | --- | --- | --- | --- | --- | --- | --- | --- | --- | --- |
| **Step 1** |  |  |  |  |  |  |  | |  |  |
| Age | -0.04 | 0.08 | -0.19 | 0.12 | -0.48 | 0.63 |  | |  |  |
| Sex ^a^ | -0.03 | 0.22 | -0.47 | 0.41 | -0.14 | 0.89 |  | |  |  |
| Parental education | 0.01 | 0.11 | -0.20 | 0.21 | 0.09 | 0.93 |  | |  |  |
| Family wealth | 0.14 | 0.06 | 0.03 | 0.25 | 2.51 | 0.01 | 0.03 | | 0.03 | 2.29 |
| **Step 2** |  |  |  |  |  |  |  | |  |  |
| Age | -0.04 | 0.08 | -0.20 | 0.11 | -0.54 | 0.59 |  | |  |  |
| Sex | -0.11 | 0.23 | -0.55 | 0.33 | -0.49 | 0.62 |  | |  |  |
| Parental education | 0.02 | 0.10 | -0.18 | 0.23 | 0.20 | 0.84 |  | |  |  |
| Family wealth | 0.12 | 0.06 | 0.01 | 0.24 | 2.17 | 0.03 |  | |  |  |
| Autonomy-supportive parenting | 0.13 | 0.14 | -0.16 | 0.41 | 0.88 | 0.38 |  | |  |  |
| Narcissism | 0.46 | 0.23 | 0.02 | 0.91 | 2.05 | 0.04 | 0.06 | | 0.03 | 3.07^**^ |
| **Step 3** |  |  |  |  |  |  |  | |  |  |
| Age | -0.05 | 0.08 | -0.21 | 0.10 | -0.67 | 0.50 |  | |  |  |
| Sex | -0.04 | 0.22 | -0.48 | 0.40 | -0.19 | 0.85 |  | |  |  |
| Parental education | -0.02 | 0.10 | -0.22 | 0.19 | -0.16 | 0.88 |  | |  |  |
| Family wealth | 0.14 | 0.06 | 0.03 | 0.25 | 2.41 | 0.02 |  | |  |  |
| Father autonomy support | 0.20 | 0.15 | -0.08 | 0.49 | 1.39 | 0.16 |  | |  |  |
| Narcissism | 0.45 | 0.22 | 0.01 | 0.89 | 2.02 | 0.04 |  | |  |  |
| Autonomy-supportive parenting X Narcissism | 0.27 | 0.10 | 0.07 | 0.47 | 2.64 | 0.01 | 0.09 | | 0.03 | 3.72^**^ |

*Note*. ^a^ coded as 0 = girls and 1 = boys.

^*^ *p* < .05, ^***^ *p* < .001.

**Figure S1.** *Johnson–Neyman regions of significance in Study 1* (*N* = 318)

**
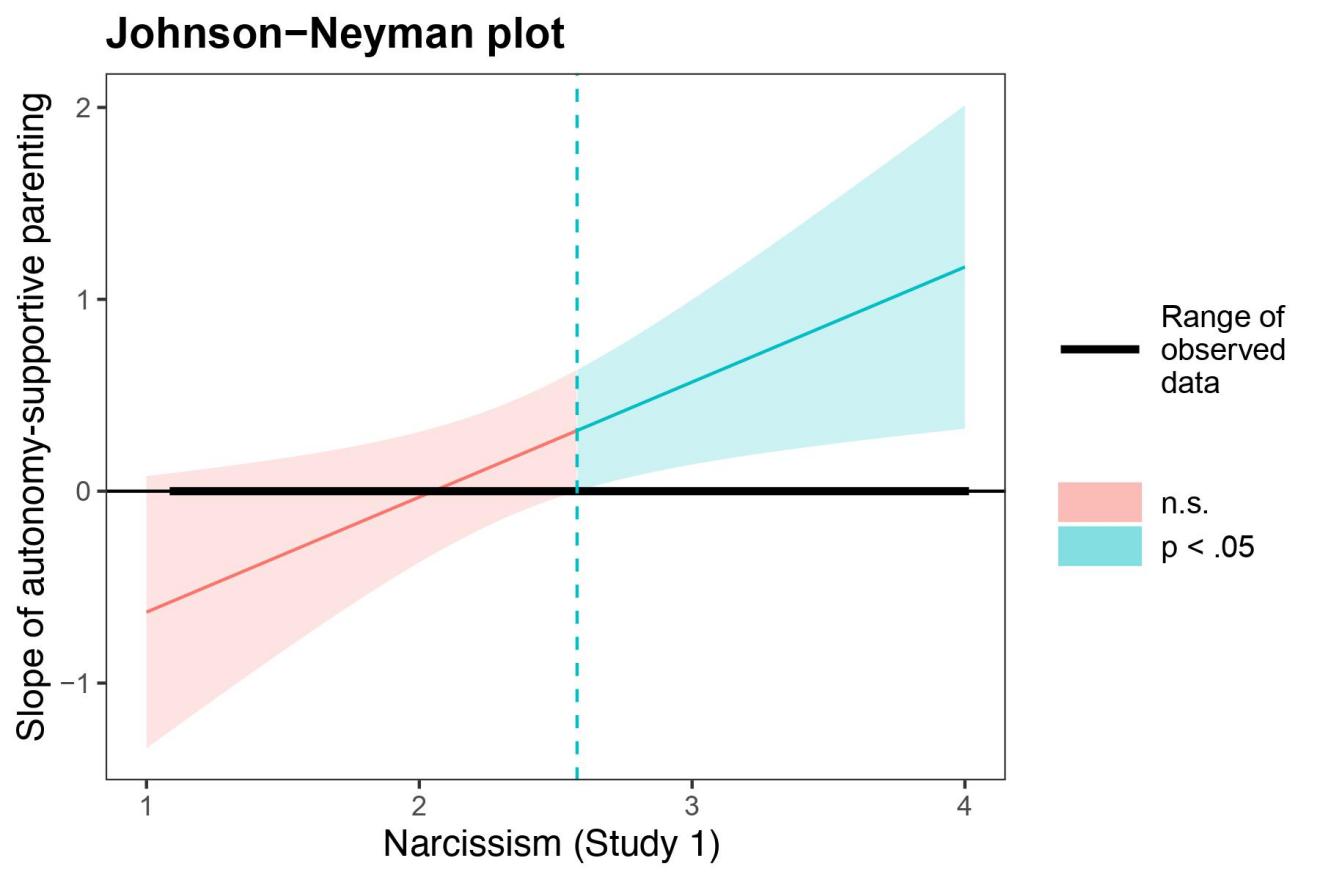
**

**Figure S2.** *The moderating role of narcissism in the association between father autonomy support and adolescents’ prosocial behavior in Study 1* (*N* = 318)

**
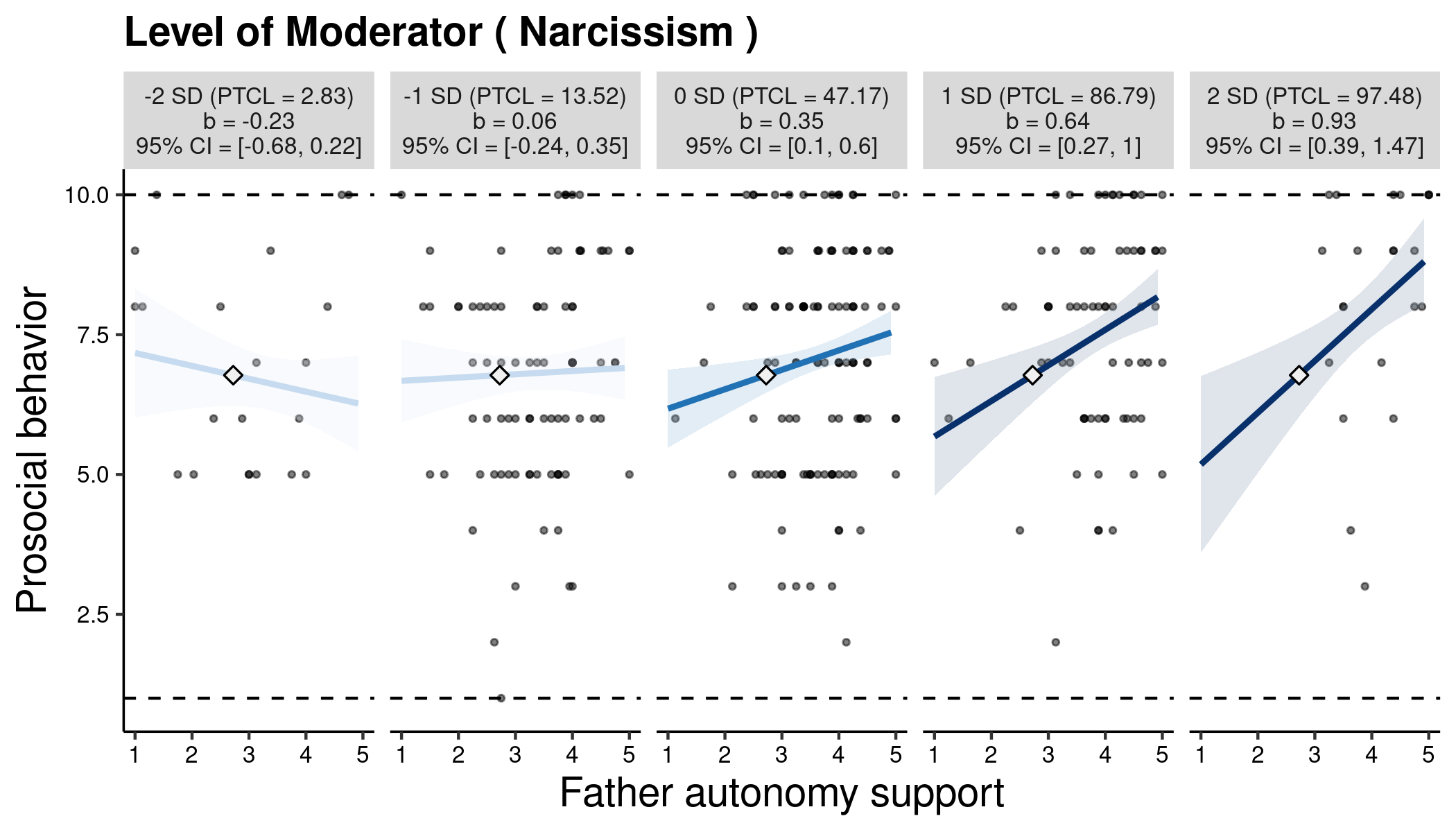
**

**Figure S3.** *The moderating role of narcissism in the association between mother autonomy support and adolescents’ prosocial behavior in Study 1* (*N* = 318)

**
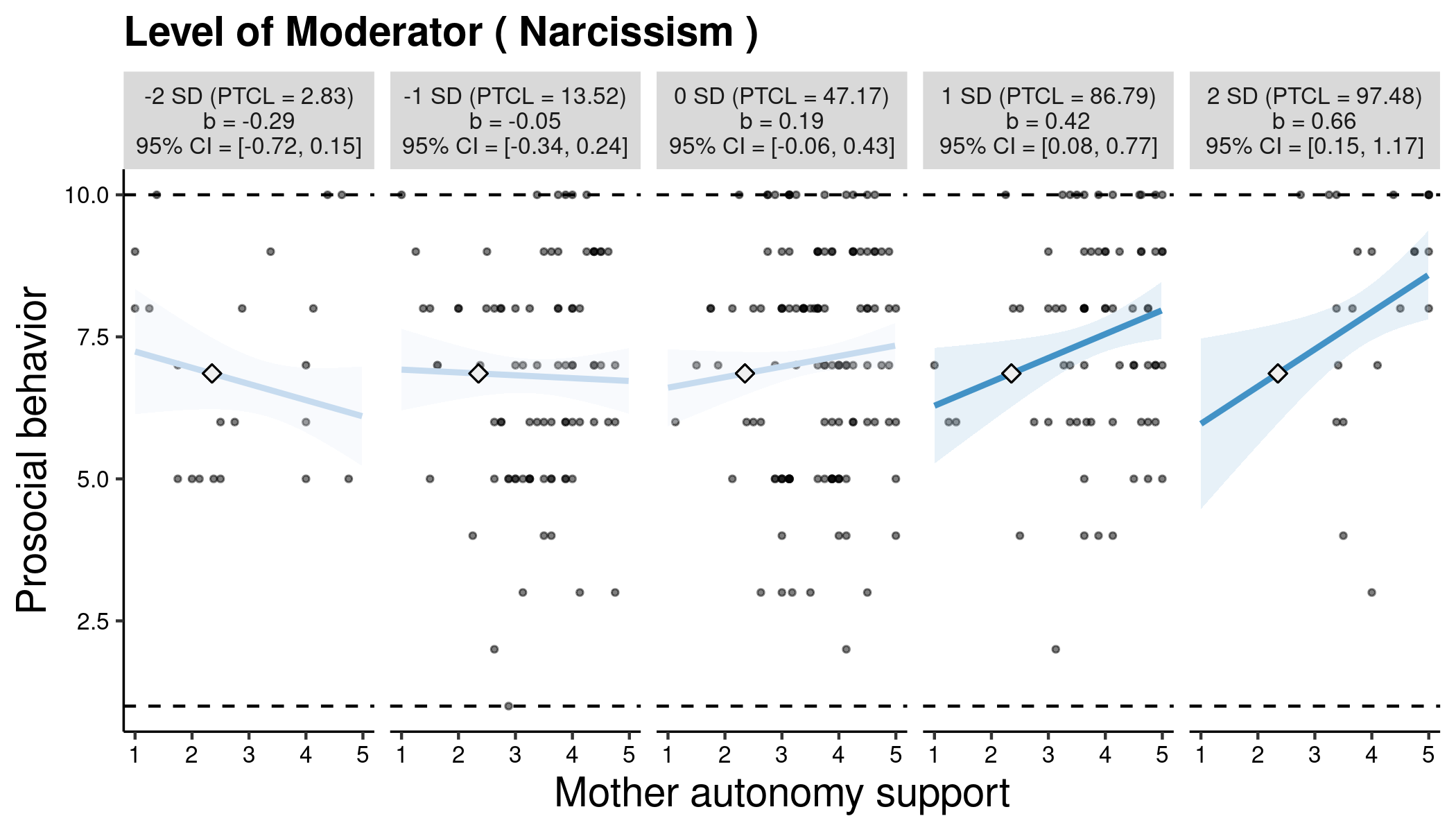
**

**Study 2**

**Table S7**. *Descriptive statistics, correlations, and inter-item reliabilities for scales used in Study 2* (*N* = 2,098)

| Variables | 1 | 2 | 3 | 4 | 5 | 6 | 7 | 8 |
| --- | --- | --- | --- | --- | --- | --- | --- | --- |
| 1. Prosocial behavior | — |  |  |  |  |  |  |  |
| 2. Autonomy-supportive parenting | 0.19^***^ | — |  |  |  |  |  |  |
| 3. Narcissism | 0.26^***^ | 0.03 | — |  |  |  |  |  |
| 4.Age | 0.01 | 0.08^***^ | -0.01 | — |  |  |  |  |
| 5. Sex ^a^ | -0.08^***^ | -0.06^**^ | 0.13^***^ | -0.01 | — |  |  |  |
| 6. Parental education | 0.07^**^ | 0.04 | 0.09^***^ | 0.08^**^ | 0.01 | — |  |  |
| 7. Family wealth | 0.06^**^ | 0.03 | 0.09^***^ | 0.07^**^ | 0.02 | 0.49^***^ | — |  |
| 8. Social desirability | 0.26^***^ | 0.12^***^ | -0.03 | -0.06^**^ | -0.03 | -0.03 | -0.05^*^ | — |
| Mean | 5.64 | 3.81 | 2.98 | 15.70 | — | 3.69 | 3.86 | 4.30 |
| *SD* | 0.83 | 0.73 | 0.68 | 1.55 | — | 1.19 | 1.05 | 0.33 |
| Minimum | 2.14 | 1.00 | 1.00 | 13.00 | 0.00 | 2.00 | 1.00 | 2.88 |
| Maximum | 7.00 | 5.00 | 5.00 | 18.00 | 1.00 | 6.00 | 7.00 | 5.88 |
| Skewness | -0.49 | -0.78 | 0.38 | -0.35 | 0.24 | 0.34 | 0.40 | 0.24 |
| Kurtosis | 0.03 | 1.17 | 0.22 | -1.16 | -1.95 | -0.47 | 1.04 | 0.86 |
| α/ϖ | 0.84/0.85 | 0.90/0.90 | 0.70/0.72 | — | — | — | — | 0.87/0.87 |

*Note*. ^a^ coded as 0 = girls and 1 = boys.

^*^ *p* < .05, ^**^ *p* < .01, ^***^ *p* < .001.

**Table S8.** *Hierarchical regression analysis predicting prosocial behavior in Study 2 with additionally including teacher autonomy support* (*N* = 2,098)

|  | *b* | *b SE* | 95% CI for *b* | | *β* | *t* | *p* | *R*^2^ | △*R*^2^ | | △*F* |
| --- | --- | --- | --- | --- | --- | --- | --- | --- | --- | --- | --- |
| **Step 1** |  |  |  |  |  |  |  |  | |  |  |
| Age | 0.01 | 0.01 | -0.02 | 0.03 | 0.01 | 0.59 | 0.55 |  | |  |  |
| Sex ^a^ | 0.13 | 0.04 | 0.06 | 0.20 | 0.08 | 3.63 | < .001 |  | |  |  |
| Parental education | 0.04 | 0.02 | 0.00 | 0.07 | 0.05 | 2.10 | 0.04 |  | |  |  |
| Family wealth | 0.04 | 0.02 | 0.00 | 0.07 | 0.05 | 1.94 | 0.05 |  | |  |  |
| Social desirability | 0.66 | 0.05 | 0.56 | 0.77 | 0.26 | 12.39 | < .001 | 0.08 | | 0.08 | 36.31^***^ |
| **Step 2** |  |  |  |  |  |  |  |  | |  |  |
| Age | 0.00 | 0.01 | -0.02 | 0.02 | 0.00 | -0.10 | 0.92 |  | |  |  |
| Sex | 0.19 | 0.03 | 0.13 | 0.25 | 0.23 | 5.82 | < .001 |  | |  |  |
| Parental education | 0.02 | 0.02 | -0.01 | 0.05 | 0.03 | 1.56 | 0.12 |  | |  |  |
| Family wealth | 0.03 | 0.02 | -0.01 | 0.06 | 0.04 | 1.62 | 0.11 |  | |  |  |
| Social desirability | 0.58 | 0.05 | 0.48 | 0.67 | 0.23 | 11.72 | < .001 |  | |  |  |
| Autonomy-supportive parenting | 0.09 | 0.02 | 0.04 | 0.13 | 0.08 | 3.93 | < .001 |  | |  |  |
| Teacher autonomy support | 0.28 | 0.02 | 0.24 | 0.32 | 0.27 | 13.46 | < .001 |  | |  |  |
| Narcissism | 0.31 | 0.02 | 0.27 | 0.36 | 0.26 | 13.15 | < .001 | 0.24 | | 0.16 | 145.34^***^ |
| **Step 3** |  |  |  |  |  |  |  |  | |  |  |
| Age | 0.00 | 0.01 | -0.02 | 0.02 | 0.00 | -0.11 | 0.91 |  | |  |  |
| Sex | 0.19 | 0.03 | 0.13 | 0.25 | 0.23 | 5.93 | < .001 |  | |  |  |
| Parental education | 0.02 | 0.02 | -0.01 | 0.05 | 0.03 | 1.58 | 0.12 |  | |  |  |
| Family wealth | 0.03 | 0.02 | 0.00 | 0.06 | 0.04 | 1.73 | 0.08 |  | |  |  |
| Social desirability | 0.57 | 0.05 | 0.47 | 0.66 | 0.22 | 11.62 | < .001 |  | |  |  |
| Autonomy-supportive parenting | -0.37 | 0.10 | -0.56 | -0.18 | 0.06 | -3.81 | < .001 |  | |  |  |
| Teacher autonomy support | 0.42 | 0.09 | 0.24 | 0.59 | 0.27 | 4.65 | < .001 |  | |  |  |
| Narcissism | -0.08 | 0.14 | -0.36 | 0.19 | 0.25 | -0.61 | 0.54 |  | |  |  |
| Autonomy-supportive parenting X Narcissism | 0.15 | 0.03 | 0.09 | 0.21 | 0.09 | 4.88 | < .001 |  | |  |  |
| Teacher autonomy support X Narcissism | -0.05 | 0.03 | -0.10 | 0.01 | -0.03 | -1.62 | 0.11 | 0.25 | | 0.01 | 11.98^***^ |

*Note*. ^a^ coded as 0 = girls and 1 = boys.

^***^ *p* < .001.

**Table S9.** *Hierarchical regression analysis predicting altruistic behavior subscale in Study 2* (*N* = 2,098)

|  | *b* | *b SE* | 95% CI for *b* | | *β* | *t* | *p* | *R*^2^ | △*R*^2^ | | △*F* |
| --- | --- | --- | --- | --- | --- | --- | --- | --- | --- | --- | --- |
| **Step 1** |  |  |  |  |  |  |  |  | |  |  |
| Age | 0.03 | 0.01 | 0.01 | 0.06 | 0.05 | 2.49 | 0.01 |  | |  |  |
| Sex ^a^ | 0.13 | 0.04 | 0.05 | 0.21 | 0.14 | 3.21 | 0.00 |  | |  |  |
| Parental education | 0.03 | 0.02 | -0.01 | 0.06 | 0.03 | 1.37 | 0.17 |  | |  |  |
| Family wealth | 0.06 | 0.02 | 0.02 | 0.11 | 0.07 | 2.81 | 0.01 |  | |  |  |
| Social desirability | 0.71 | 0.06 | 0.59 | 0.83 | 0.24 | 11.48 | < .001 | 0.07 | | 0.07 | 32.56^***^ |
| **Step 2** |  |  |  |  |  |  |  |  | |  |  |
| Age | 0.03 | 0.01 | 0.01 | 0.06 | 0.05 | 2.45 | 0.01 |  | |  |  |
| Sex | 0.18 | 0.04 | 0.10 | 0.26 | 0.19 | 4.59 | < .001 |  | |  |  |
| Parental education | 0.01 | 0.02 | -0.03 | 0.05 | 0.01 | 0.61 | 0.54 |  | |  |  |
| Family wealth | 0.05 | 0.02 | 0.00 | 0.09 | 0.05 | 2.17 | 0.03 |  | |  |  |
| Social desirability | 0.68 | 0.06 | 0.57 | 0.80 | 0.23 | 11.46 | < .001 |  | |  |  |
| Autonomy-supportive parenting | 0.14 | 0.03 | 0.09 | 0.20 | 0.11 | 5.44 | < .001 |  | |  |  |
| Narcissism | 0.36 | 0.03 | 0.31 | 0.42 | 0.26 | 12.56 | < .001 | 0.15 | | 0.08 | 96.82^***^ |
| **Step 3** |  |  |  |  |  |  |  |  | |  |  |
| Age | 0.03 | 0.01 | 0.01 | 0.05 | 0.05 | 2.42 | 0.02 |  | |  |  |
| Sex | 0.18 | 0.04 | 0.11 | 0.26 | 0.19 | 4.70 | < .001 |  | |  |  |
| Parental education | 0.01 | 0.02 | -0.02 | 0.05 | 0.01 | 0.62 | 0.54 |  | |  |  |
| Family wealth | 0.05 | 0.02 | 0.01 | 0.09 | 0.05 | 2.26 | 0.02 |  | |  |  |
| Social desirability | 0.68 | 0.06 | 0.56 | 0.79 | 0.23 | 11.38 | < .001 |  | |  |  |
| Autonomy-supportive parenting | -0.23 | 0.11 | -0.45 | -0.01 | 0.10 | -2.02 | 0.04 |  | |  |  |
| Narcissism | -0.10 | 0.14 | -0.38 | 0.17 | 0.25 | -0.73 | 0.46 |  | |  |  |
| Autonomy-supportive parenting X Narcissism | 0.12 | 0.04 | 0.05 | 0.19 | 0.06 | 3.39 | < .001 | 0.16 | | 0.01 | 11.51^***^ |

*Note*. ^a^ coded as 0 = girls and 1 = boys.

^*^ *p* < .05, ^**^ *p* < .01, ^***^ *p* < .001.

**Table S10.** *Hierarchical regression analysis predicting public good subscale in Study 2* (*N* = 2,098)

|  | *b* | *b SE* | 95% CI for *b* | | *β* | *t* | *p* | *R*^2^ | △*R*^2^ | | △*F* |
| --- | --- | --- | --- | --- | --- | --- | --- | --- | --- | --- | --- |
| **Step 1** |  |  |  |  |  |  |  |  | |  |  |
| Age | -0.01 | 0.01 | -0.04 | 0.01 | -0.02 | -0.84 | 0.40 |  | |  |  |
| Sex ^a^ | 0.17 | 0.04 | 0.09 | 0.24 | 0.18 | 4.29 | < .001 |  | |  |  |
| Parental education | 0.02 | 0.02 | -0.02 | 0.06 | 0.02 | 0.99 | 0.32 |  | |  |  |
| Family wealth | 0.02 | 0.02 | -0.02 | 0.06 | 0.02 | 0.84 | 0.40 |  | |  |  |
| Social desirability | 0.57 | 0.06 | 0.46 | 0.69 | 0.21 | 9.68 | < .001 | 0.05 | | 0.05 | 23.61^***^ |
| **Step 2** |  |  |  |  |  |  |  |  | |  |  |
| Age | -0.01 | 0.01 | -0.04 | 0.01 | -0.02 | -1.18 | 0.24 |  | |  |  |
| Sex | 0.20 | 0.04 | 0.13 | 0.28 | 0.22 | 5.36 | < .001 |  | |  |  |
| Parental education | 0.00 | 0.02 | -0.03 | 0.04 | 0.01 | 0.25 | 0.80 |  | |  |  |
| Family wealth | 0.00 | 0.02 | -0.04 | 0.04 | 0.00 | 0.19 | 0.85 |  | |  |  |
| Social desirability | 0.53 | 0.06 | 0.42 | 0.65 | 0.19 | 9.32 | < .001 |  | |  |  |
| Autonomy-supportive parenting | 0.19 | 0.03 | 0.13 | 0.24 | 0.15 | 7.21 | < .001 |  | |  |  |
| Narcissism | 0.31 | 0.03 | 0.25 | 0.36 | 0.23 | 10.99 | < .001 | 0.13 | | 0.08 | 89.82^***^ |
| **Step 3** |  |  |  |  |  |  |  |  | |  |  |
| Age | -0.01 | 0.01 | -0.04 | 0.01 | -0.03 | -1.24 | 0.22 |  | |  |  |
| Sex | 0.21 | 0.04 | 0.13 | 0.28 | 0.23 | 5.49 | < .001 |  | |  |  |
| Parental education | 0.00 | 0.02 | -0.03 | 0.04 | 0.01 | 0.26 | 0.80 |  | |  |  |
| Family wealth | 0.01 | 0.02 | -0.03 | 0.05 | 0.01 | 0.31 | 0.75 |  | |  |  |
| Social desirability | 0.52 | 0.06 | 0.41 | 0.64 | 0.19 | 9.20 | < .001 |  | |  |  |
| Autonomy-supportive parenting | -0.30 | 0.11 | -0.51 | -0.08 | 0.13 | -2.73 | 0.01 |  | |  |  |
| Narcissism | -0.29 | 0.13 | -0.56 | -0.03 | 0.22 | -2.18 | 0.03 |  | |  |  |
| Autonomy-supportive parenting X Narcissism | 0.16 | 0.03 | 0.09 | 0.22 | 0.09 | 4.55 | < .001 | 0.14 | | 0.01 | 20.70^***^ |

*Note*. ^a^ coded as 0 = girls and 1 = boys.

^*^ *p* < .05, ^**^ *p* < .01, ^***^ *p* < .001.

**Table S11.** *Hierarchical regression analysis predicting relational behavior subscale in Study 2* (*N* = 2,098)

|  | *b* | *b SE* | 95% CI for *b* | | *β* | *t* | *p* | *R*^2^ | △*R*^2^ | | △*F* |
| --- | --- | --- | --- | --- | --- | --- | --- | --- | --- | --- | --- |
| **Step 1** |  |  |  |  |  |  |  |  | |  |  |
| Age | -0.01 | 0.01 | -0.04 | 0.01 | -0.02 | -1.14 | 0.26 |  | |  |  |
| Sex ^a^ | 0.05 | 0.04 | -0.03 | 0.13 | 0.05 | 1.23 | 0.22 |  | |  |  |
| Parental education | 0.07 | 0.02 | 0.03 | 0.11 | 0.09 | 3.70 | < .001 |  | |  |  |
| Family wealth | 0.03 | 0.02 | -0.01 | 0.07 | 0.04 | 1.51 | 0.13 |  | |  |  |
| Social desirability | 0.68 | 0.06 | 0.57 | 0.80 | 0.24 | 11.45 | < .001 | 0.07 | | 0.07 | 31.84^***^ |
| **Step 2** |  |  |  |  |  |  |  |  | |  |  |
| Age | -0.02 | 0.01 | -0.04 | 0.01 | -0.03 | -1.36 | 0.18 |  | |  |  |
| Sex | 0.10 | 0.04 | 0.02 | 0.17 | 0.10 | 2.54 | 0.01 |  | |  |  |
| Parental education | 0.05 | 0.02 | 0.02 | 0.09 | 0.07 | 2.99 | 0.00 |  | |  |  |
| Family wealth | 0.02 | 0.02 | -0.02 | 0.06 | 0.02 | 0.81 | 0.42 |  | |  |  |
| Social desirability | 0.65 | 0.06 | 0.54 | 0.77 | 0.23 | 11.38 | < .001 |  | |  |  |
| Autonomy-supportive parenting | 0.16 | 0.03 | 0.11 | 0.21 | 0.13 | 6.20 | < .001 |  | |  |  |
| Narcissism | 0.36 | 0.03 | 0.30 | 0.41 | 0.26 | 12.88 | < .001 | 0.16 | | 0.09 | 105.84^***^ |
| **Step 3** |  |  |  |  |  |  |  |  | |  |  |
| Age | -0.02 | 0.01 | -0.04 | 0.01 | -0.03 | -1.44 | 0.15 |  | |  |  |
| Sex | 0.10 | 0.04 | 0.03 | 0.18 | 0.11 | 2.71 | 0.01 |  | |  |  |
| Parental education | 0.05 | 0.02 | 0.02 | 0.09 | 0.07 | 3.02 | 0.00 |  | |  |  |
| Family wealth | 0.02 | 0.02 | -0.02 | 0.06 | 0.02 | 0.97 | 0.33 |  | |  |  |
| Social desirability | 0.64 | 0.06 | 0.53 | 0.75 | 0.23 | 11.27 | < .001 |  | |  |  |
| Autonomy-supportive parenting | -0.48 | 0.11 | -0.69 | -0.27 | 0.11 | -4.39 | < .001 |  | |  |  |
| Narcissism | -0.43 | 0.13 | -0.70 | -0.17 | 0.26 | -3.22 | 0.00 |  | |  |  |
| Autonomy-supportive parenting X Narcissism | 0.21 | 0.03 | 0.14 | 0.27 | 0.11 | 6.02 | < .001 | 0.17 | | 0.01 | 36.20^***^ |

*Note*. ^a^ coded as 0 = girls and 1 = boys.

^*^ *p* < .05, ^**^ *p* < .01, ^***^ *p* < .001.

**Table S12.** *Hierarchical regression analysis predicting trait prosociality subscale* *in Study 2* (*N* = 2,098)

|  | *b* | *b SE* | 95% CI for *b* | | *β* | *t* | *p* | *R*^2^ | △*R*^2^ | | △*F* |
| --- | --- | --- | --- | --- | --- | --- | --- | --- | --- | --- | --- |
| **Step 1** |  |  |  |  |  |  |  |  | |  |  |
| Age | 0.01 | 0.01 | -0.02 | 0.03 | 0.01 | 0.53 | 0.60 |  | |  |  |
| Sex ^a^ | 0.15 | 0.04 | 0.08 | 0.22 | 0.18 | 4.19 | < .001 |  | |  |  |
| Parental education | 0.03 | 0.02 | 0.00 | 0.06 | 0.04 | 1.75 | 0.08 |  | |  |  |
| Family wealth | 0.04 | 0.02 | 0.00 | 0.08 | 0.05 | 1.86 | 0.06 |  | |  |  |
| Social desirability | 0.64 | 0.06 | 0.53 | 0.75 | 0.24 | 11.53 | < .001 | 0.07 | | 0.07 | 32.63^***^ |
| **Step 2** |  |  |  |  |  |  |  |  | |  |  |
| Age | 0.00 | 0.01 | -0.02 | 0.02 | 0.00 | 0.18 | 0.86 |  | |  |  |
| Sex | 0.18 | 0.04 | 0.11 | 0.25 | 0.21 | 5.16 | < .001 |  | |  |  |
| Parental education | 0.02 | 0.02 | -0.02 | 0.05 | 0.02 | 1.07 | 0.29 |  | |  |  |
| Family wealth | 0.02 | 0.02 | -0.01 | 0.06 | 0.03 | 1.25 | 0.21 |  | |  |  |
| Social desirability | 0.60 | 0.05 | 0.49 | 0.70 | 0.23 | 11.16 | < .001 |  | |  |  |
| Autonomy-supportive parenting | 0.18 | 0.02 | 0.13 | 0.23 | 0.15 | 7.43 | < .001 |  | |  |  |
| Narcissism | 0.26 | 0.03 | 0.21 | 0.32 | 0.21 | 10.18 | < .001 | 0.14 | | 0.07 | 82.86^***^ |
| **Step 3** |  |  |  |  |  |  |  |  | |  |  |
| Age | 0.00 | 0.01 | -0.02 | 0.02 | 0.00 | 0.14 | 0.89 |  | |  |  |
| Sex | 0.19 | 0.04 | 0.12 | 0.25 | 0.22 | 5.26 | < .001 |  | |  |  |
| Parental education | 0.02 | 0.02 | -0.01 | 0.05 | 0.03 | 1.08 | 0.28 |  | |  |  |
| Family wealth | 0.03 | 0.02 | -0.01 | 0.06 | 0.03 | 1.36 | 0.18 |  | |  |  |
| Social desirability | 0.59 | 0.05 | 0.49 | 0.70 | 0.23 | 11.06 | < .001 |  | |  |  |
| Autonomy-supportive parenting | -0.19 | 0.10 | -0.39 | 0.01 | 0.14 | -1.85 | 0.07 |  | |  |  |
| Narcissism | -0.19 | 0.13 | -0.44 | 0.06 | 0.21 | -1.51 | 0.13 |  | |  |  |
| Autonomy-supportive parenting X Narcissism | 0.12 | 0.03 | 0.06 | 0.18 | 0.07 | 3.69 | < .001 | 0.15 | | 0.01 | 13.62^***^ |

*Note*. ^a^ coded as 0 = girls and 1 = boys.

^*^ *p* < .05, ^**^ *p* < .01, ^***^ *p* < .001.

**Figure S4.** *Johnson–Neyman regions of significance in Study 2* (*N* = 2,098)

**
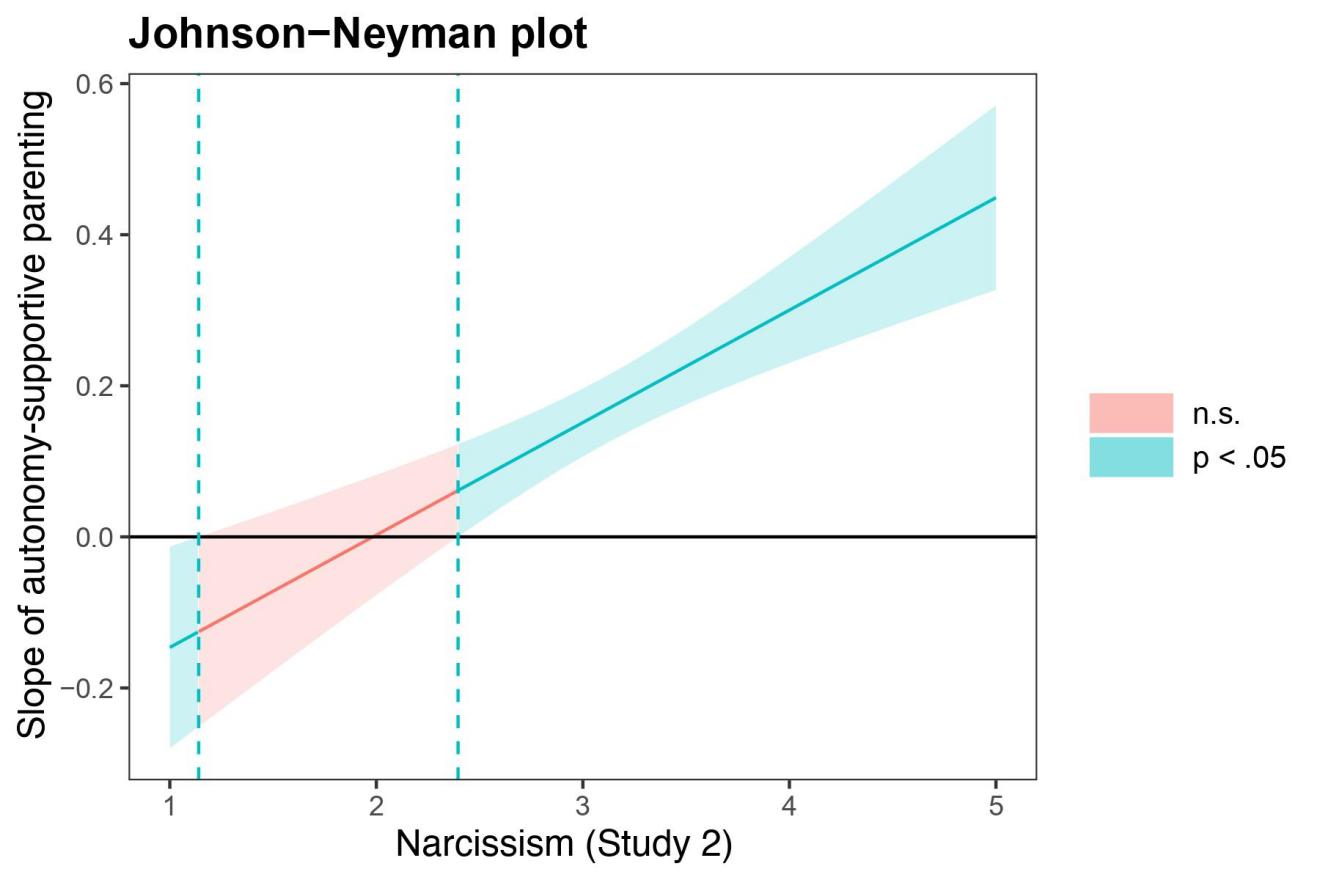
**

**Figure S5.** *The moderating role of narcissism in the association between autonomy-supportive parenting and adolescents’ altruistic behavior in Study 2* (*N* = 2,098)


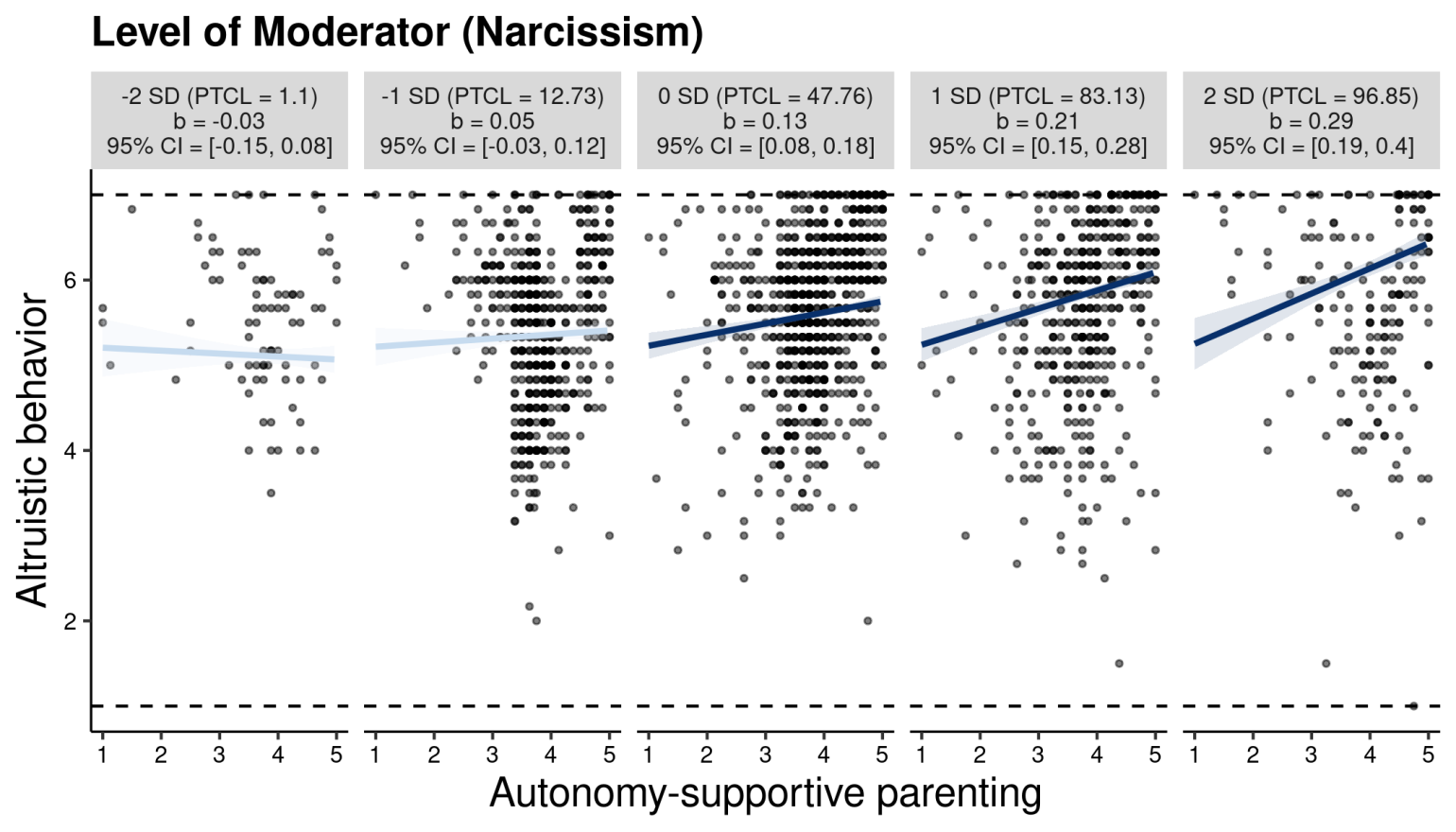


**Figure S6.** *The moderating role of narcissism in the association between autonomy-supportive parenting and public good in Study 2* (*N* = 2,098)


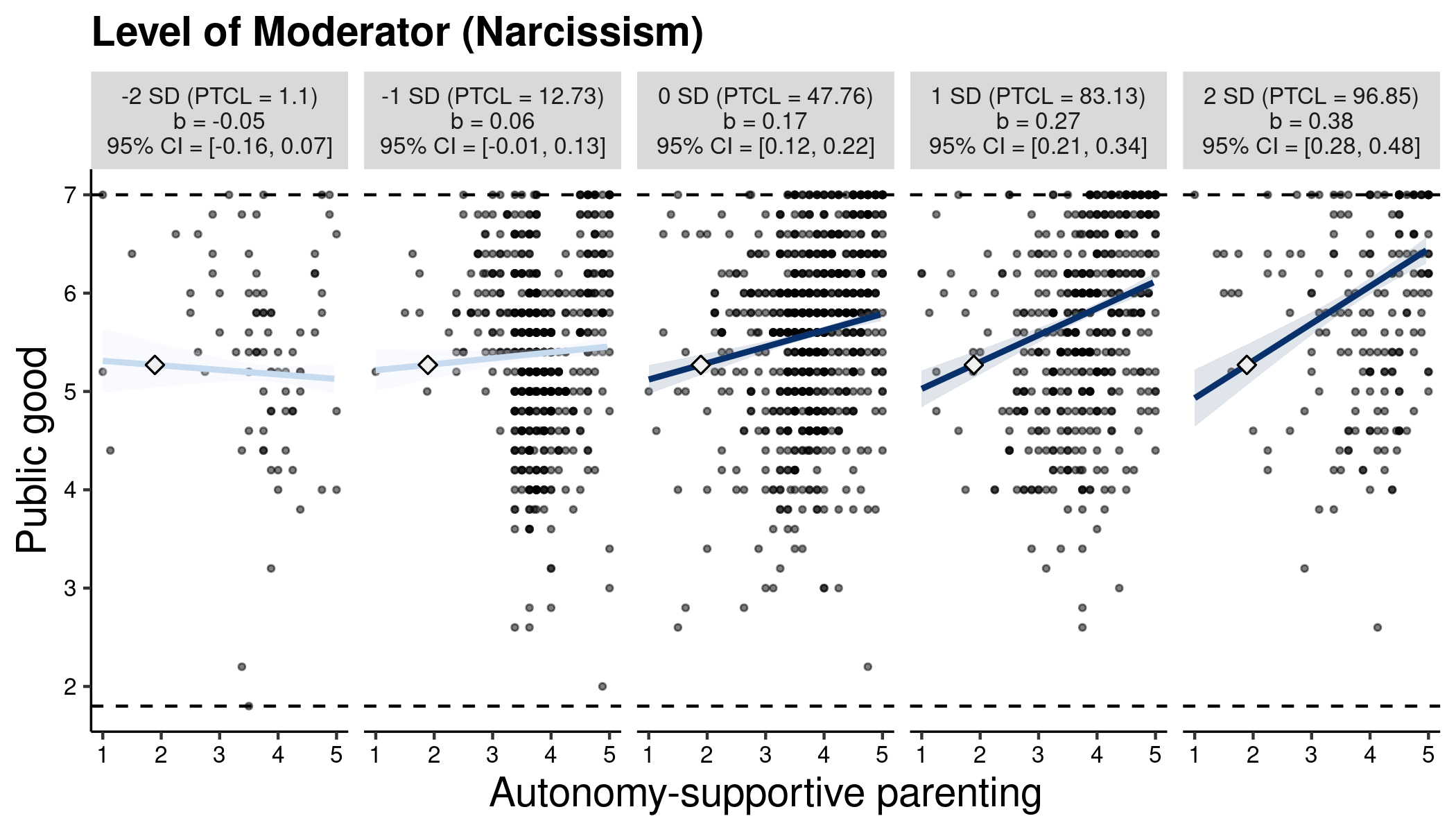


**Figure S7.** *The moderating role of narcissism in the association between autonomy-supportive parenting and adolescents’ relational behavior in Study 2* (*N* = 2,098)


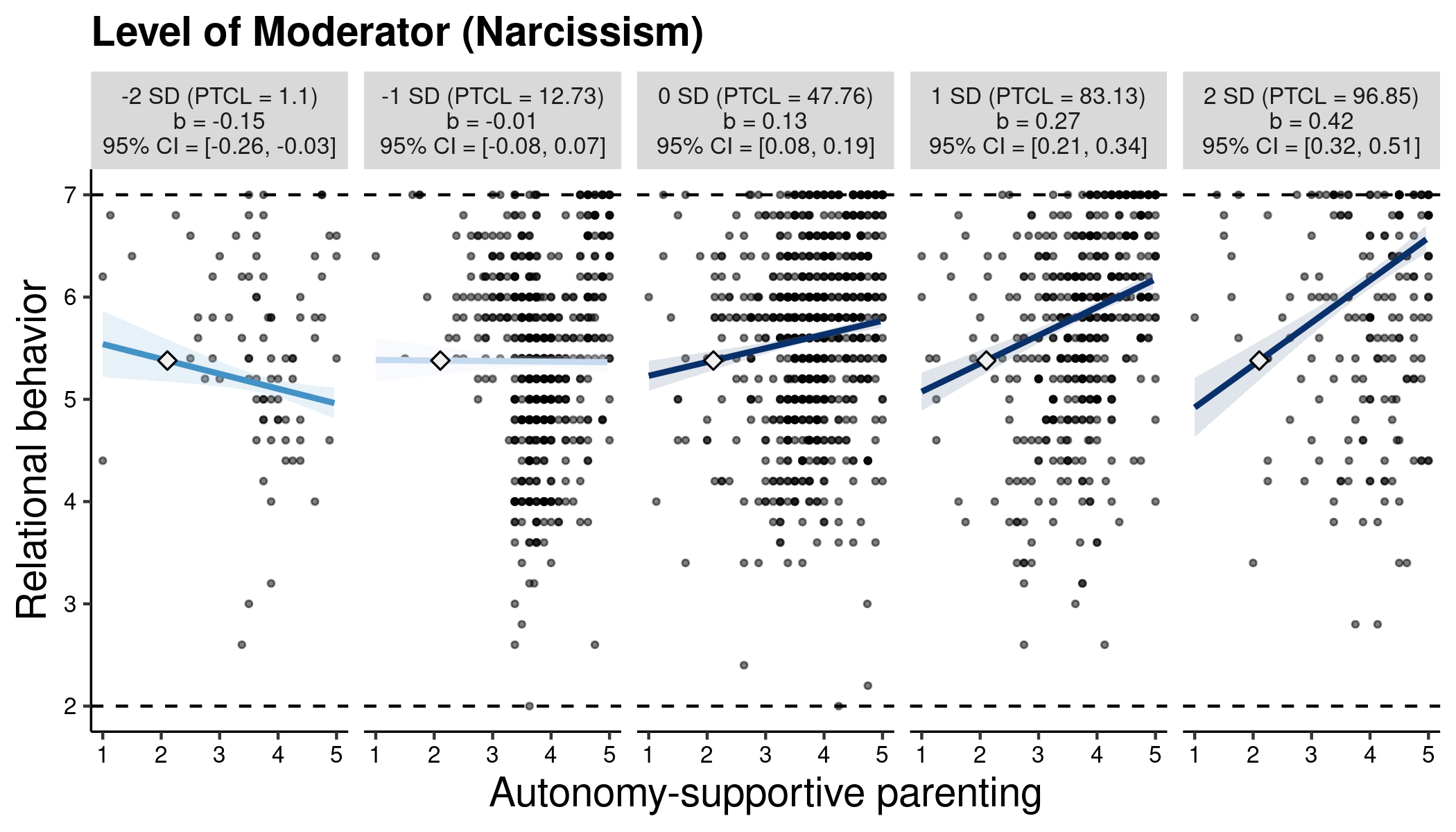


**Figure S8.** *The moderating role of narcissism in the association between autonomy-supportive parenting and adolescents’ prosocial behavior in Study 2* (*N* = 2,098)


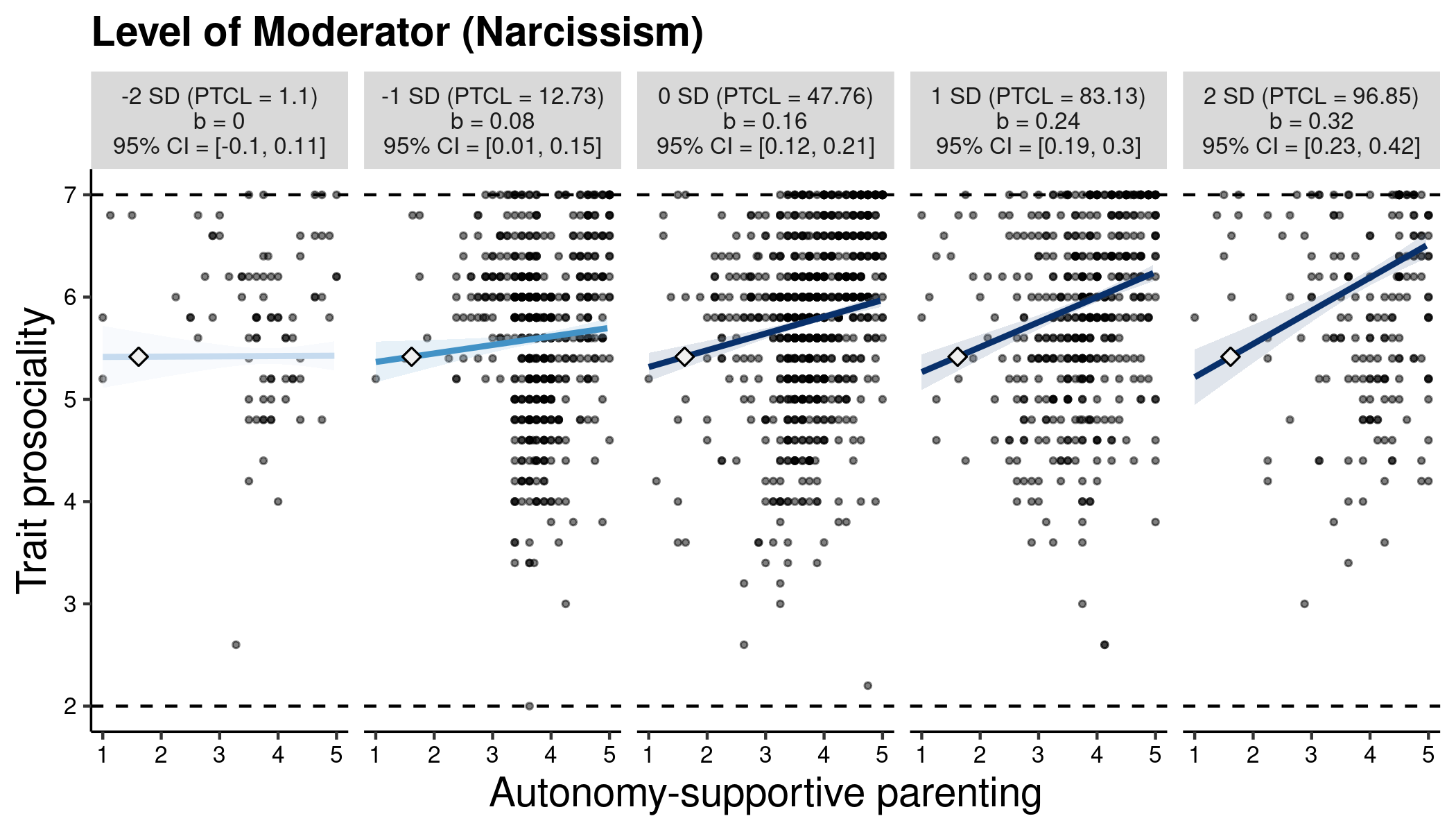


**Study 3**

**Table S13**. *Descriptive statistics, correlations, and inter-item reliabilities for scales used in Study 3* (*N* = 629)

| Variables | 1 | 2 | 3 | 4 | 5 | 6 | 7 | 8 | 9 |
| --- | --- | --- | --- | --- | --- | --- | --- | --- | --- |
| 1. Prosocial behavior (Time 2) | — |  |  |  |  |  |  |  |  |
| 2. Autonomy-supportive parenting (Time 2) | 0.20^***^ | — |  |  |  |  |  |  |  |
| 3.Autonomy-supportive parenting (Time 1) | 0.14^***^ | 0.26^***^ | — |  |  |  |  |  |  |
| 4. Narcissism (Time 2) | 0.05 | 0.10^**^ | 0.02 | — |  |  |  |  |  |
| 5.Age (Time 1) | -0.06 | -0.02 | -0.12^**^ | 0.04 | — |  |  |  |  |
| 6. Sex (Time 1) ^a^ | 0.07 | 0.07 | 0.04 | -0.11^**^ | -0.09^*^ | — |  |  |  |
| 7. Parental education (Time 1) | -0.07 | -0.01 | 0.05 | 0.02 | -0.14^***^ | 0.04 | — |  |  |
| 8. Family wealth (Time 1) | 0.05 | 0.13^**^ | 0.22^***^ | 0.02 | 0.08^*^ | 0.06 | 0.07 | — |  |
| 9. Prosocial behavior (Time 1) | 0.16^***^ | 0.14^***^ | 0.24^***^ | 0.01 | -0.09^*^ | 0.08^*^ | 0.07 | 0.01 | — |
| Mean | 6.45 | 3.53 | 3.36 | 2.92 | 12.86 | 0.52 | 2.55 | 3.83 | 6.35 |
| *SD* | 2.12 | 0.84 | 0.81 | 0.50 | 1.70 | 0.50 | 0.68 | 2.00 | 2.29 |
| Minimum | 0.00 | 1.00 | 1.00 | 1.00 | 10.00 | 0.00 | 2.00 | 0.00 | 0.00 |
| Maximum | 10.00 | 5.00 | 5.00 | 5.00 | 15.00 | 1.00 | 6.00 | 9.00 | 10.00 |
| Skewness | -0.15 | -0.56 | -0.27 | -0.11 | -0.28 | -0.06 | 2.27 | -0.05 | -0.15 |
| Kurtosis | 0.07 | 0.24 | -0.09 | 0.99 | -1.32 | -2.00 | 7.04 | -0.73 | -0.47 |
| α/ϖ | 0.75/0.75 | 0.94/0.94 | 0.87/0.87 | 0.67/0.68 | — | — | — | — | 0.70/0.70 |

*Note*. ^a^ coded as 0 = girls and 1 = boys.

^*^ *p* < .05, ^**^ *p* < .01, ^***^ *p* < .001.

**Table S14.** *Hierarchical regression analysis predicting prosocial behavior in Study 3 with additionally including the changes in teacher and peer autonomy support* (*N* = 629)

|  | *b* | *b SE* | 95% CI for *b* | | *β* | *t* | *p* | *R*^2^ | △*R*^2^ | | △*F* |
| --- | --- | --- | --- | --- | --- | --- | --- | --- | --- | --- | --- |
| **Step 1** |  |  |  |  |  |  |  |  | |  |  |
| Age (Time 1) | -0.08 | 0.05 | -0.18 | 0.02 | -0.06 | -1.58 | 0.11 |  | |  |  |
| Sex (Time 1) ^a^ | 0.20 | 0.17 | -0.13 | 0.53 | 0.05 | 1.19 | 0.24 |  | |  |  |
| Parental education (Time 1) | -0.30 | 0.12 | -0.54 | -0.05 | -0.09 | -2.38 | 0.02 |  | |  |  |
| Family wealth (Time 1) | 0.06 | 0.04 | -0.03 | 0.14 | 0.05 | 1.32 | 0.19 |  | |  |  |
| Prosocial behavior (Time 1) | 0.15 | 0.04 | 0.08 | 0.22 | 0.16 | 4.06 | < .001 | 0.04 | | 0.04 | 5.52^***^ |
| **Step 2** |  |  |  |  |  |  |  |  | |  |  |
| Age (Time 1) | -0.07 | 0.05 | -0.17 | 0.03 | -0.06 | -1.42 | 0.16 |  | |  |  |
| Sex (Time 1) | 0.15 | 0.17 | -0.18 | 0.48 | 0.07 | 0.90 | 0.37 |  | |  |  |
| Parental education (Time 1) | -0.29 | 0.12 | -0.53 | -0.05 | -0.09 | -2.36 | 0.02 |  | |  |  |
| Family wealth (Time 1) | 0.03 | 0.04 | -0.05 | 0.12 | 0.03 | 0.82 | 0.41 |  | |  |  |
| Prosocial behavior (Time 1) | 0.13 | 0.04 | 0.06 | 0.20 | 0.14 | 3.59 | < .001 |  | |  |  |
| △Autonomy-supportive parenting | 0.20 | 0.09 | 0.03 | 0.38 | 0.10 | 2.29 | 0.02 |  | |  |  |
| △ Teacher autonomy support | 0.16 | 0.10 | -0.03 | 0.35 | 0.08 | 1.69 | 0.09 |  | |  |  |
| △ Peer autonomy support | 0.18 | 0.10 | -0.01 | 0.38 | 0.09 | 1.83 | 0.07 |  | |  |  |
| Narcissism (Time 2) | 0.18 | 0.17 | -0.15 | 0.50 | 0.04 | 1.06 | 0.29 | 0.08 | | 0.04 | 6.77^***^ |
| **Step 3** |  |  |  |  |  |  |  |  | |  |  |
| Age (Time 1) | -0.07 | 0.05 | -0.16 | 0.03 | -0.05 | -1.37 | 0.17 |  | |  |  |
| Sex (Time 1) | 0.17 | 0.17 | -0.16 | 0.50 | 0.04 | 1.01 | 0.31 |  | |  |  |
| Parental education (Time 1) | -0.28 | 0.12 | -0.52 | -0.04 | -0.09 | -2.32 | 0.02 |  | |  |  |
| Family wealth (Time 1) | 0.03 | 0.04 | -0.06 | 0.11 | 0.02 | 0.62 | 0.53 |  | |  |  |
| Prosocial behavior (Time 1) | 0.13 | 0.04 | 0.06 | 0.20 | 0.14 | 3.70 | < .001 |  | |  |  |
| △Autonomy-supportive parenting | -0.79 | 0.47 | -1.72 | 0.15 | 0.10 | -1.65 | 0.10 |  | |  |  |
| △ Teacher autonomy support | -0.06 | 0.55 | -1.15 | 1.02 | 0.07 | -0.11 | 0.91 |  | |  |  |
| △ Peer autonomy support | 0.58 | 0.55 | -0.49 | 1.65 | 0.09 | 1.07 | 0.29 |  | |  |  |
| Narcissism (Time 2) | 0.15 | 0.17 | -0.18 | 0.47 | 0.03 | 0.89 | 0.37 |  | |  |  |
| △Autonomy-supportive parenting X Narcissism | 0.34 | 0.16 | 0.02 | 0.66 | 0.08 | 2.11 | 0.04 |  | |  |  |
| △ Teacher autonomy support X Narcissism | 0.07 | 0.18 | -0.29 | 0.43 | 0.02 | 0.40 | 0.69 |  | |  |  |
| △ Peer autonomy support X Narcissism | -0.14 | 0.18 | -0.49 | 0.22 | -0.03 | -0.75 | 0.45 | 0.09 | | 0.01 | 1.55 |

*Note*. ^a^ coded as 0 = girls and 1 = boys. ^***^ *p* < .001.

**Figure S9.** *Johnson–Neyman regions of significance in Study 3* (*N* = 629)


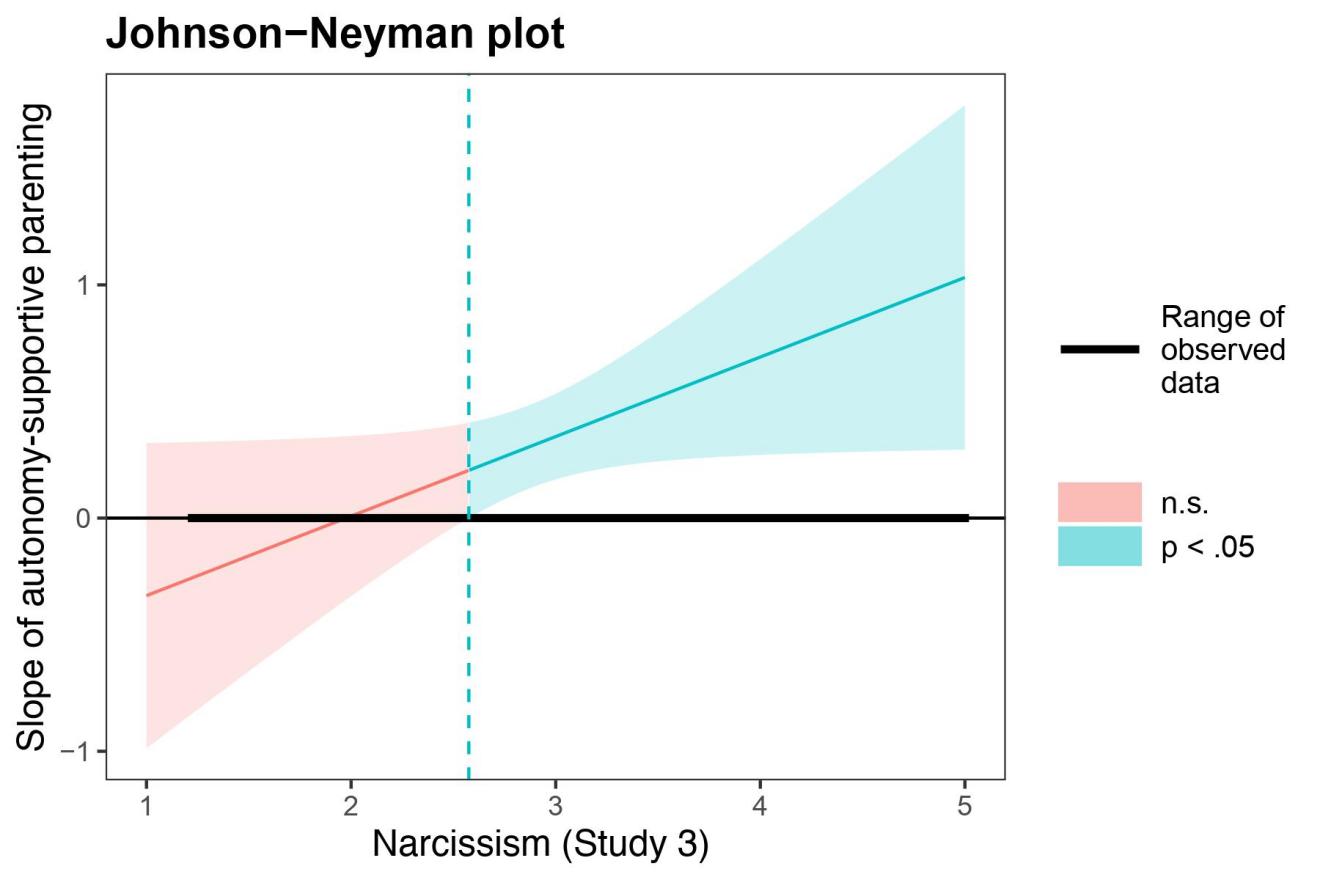


**Study 4**

**Autonomy-supportive parenting manipulation**

Autonomy-supportive condition: When deciding on matters related to me, my parents will be open to my opinions and be willing to consider those issues from my standpoint (taking the adolescent’s perspectives), and encourage me to put forward my own ideas (providing choices); when I have conflict with my parents’ perspectives, my parents will also explain why they decide to do so (providing a meaningful rationale).

Autonomy-suppressive condition: When deciding on matters related to me, my parents told me that what they chose for me was the best option. I will understand when I become more mature, and I "should" not question their decision right now.

**Table S15**. *Descriptive statistics, correlations, and inter-item reliabilities for scales used in Study 4* (*N* = 118)

| Variables | 1 | 2 | 3 | 4 | 5 | 6 | 7 |
| --- | --- | --- | --- | --- | --- | --- | --- |
| 1. Prosocial behavior | — |  |  |  |  |  |  |
| 2. Narcissism | 0.09 | — |  |  |  |  |  |
| 3.Age | -0.06 | 0.00 | — |  |  |  |  |
| 4. Sex ^a^ | -0.06 | 0.09 | 0.09 | — |  |  |  |
| 5. Parental education | -0.14 | 0.05 | -0.06 | -0.30^***^ | — |  |  |
| 6. Family wealth | 0.07 | 0.14 | 0.06 | -0.19^*^ | 0.31^***^ | — |  |
| 7. Social desirability | 0.47^***^ | 0.37^***^ | -0.12 | 0.16 | -0.06 | 0.06 | — |
| Mean | 5.34 | 2.71 | 12.42 | — | 3.14 | 3.05 | 4.79 |
| *SD* | 0.93 | 0.51 | 0.80 | — | 1.37 | 1.24 | 1.00 |
| Minimum | 2.50 | 1.33 | 10.00 | 0.00 | 2.00 | 1.00 | 1.80 |
| Maximum | 6.90 | 4.44 | 15.00 | 1.00 | 6.00 | 6.00 | 7.00 |
| Skewness | -0.51 | 0.07 | 1.05 | — | 0.99 | 0.26 | 0.01 |
| Kurtosis | 0.15 | 0.74 | 2.67 | — | -0.26 | -0.57 | 0.15 |
| α/ϖ | 0.94/0.94 | 0.72/0.74 | — | — | — | — | 0.75/0.74 |

*Note*. ^a^ coded as 0 = girls and 1 = boys.

^*^ *p* < .05, ^**^ *p* < .01, ^***^ *p* < .001.

**Figure S10.** *Johnson–Neyman regions of significance in Study 4* (*N* = 118)


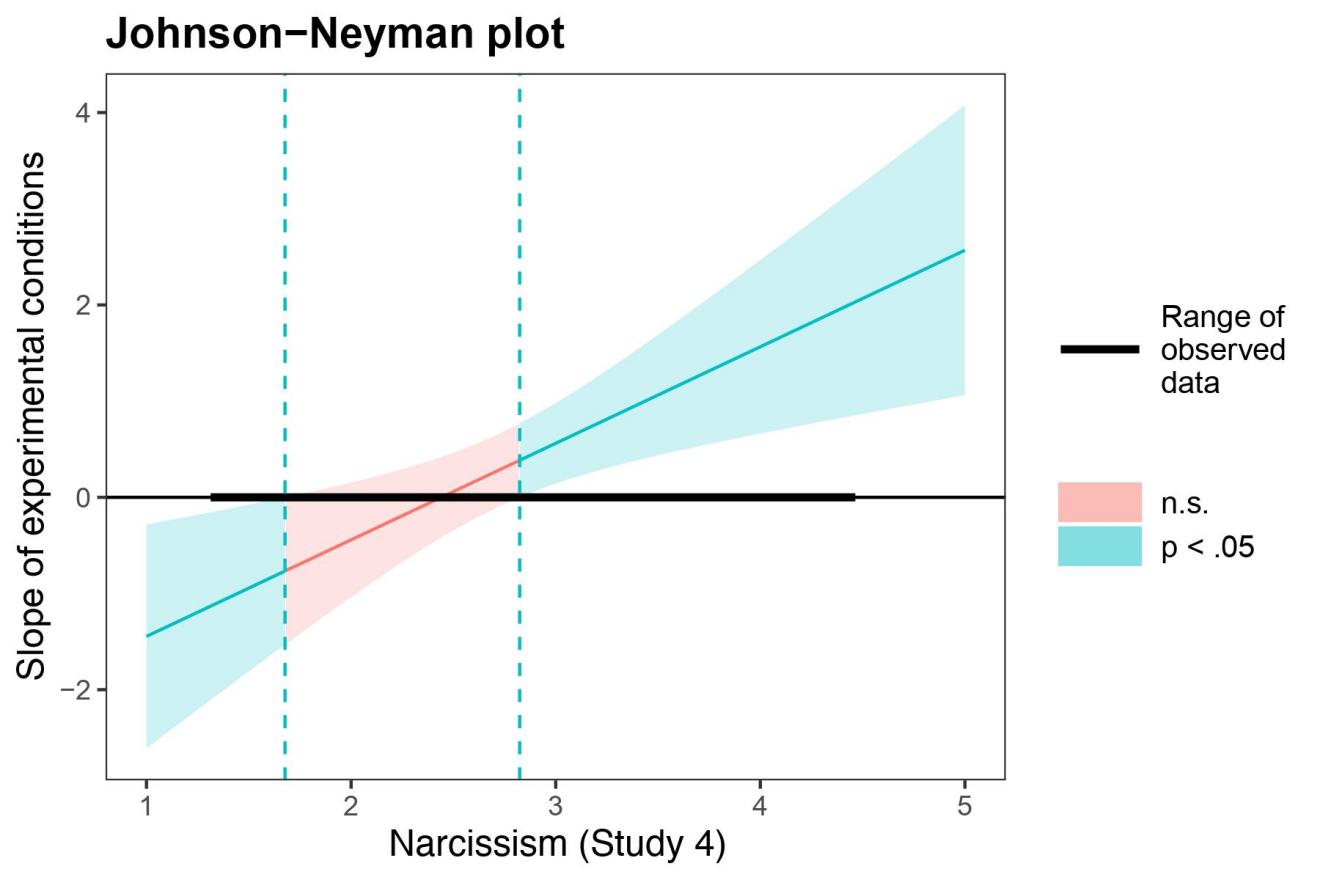

Supplement: Supplementary file 1 — Supplementary Information [file 10964_2023_1933_MOESM1_ESM.docx]
